# Supplementary material for: Comparative efficacy and safety of statin and fibrate monotherapy: A systematic review and meta-analysis of head-to-head randomized controlled trials
Source: PLoS One. 2021 Feb 9;16(2):e0246480. doi: 10.1371/journal.pone.0246480 (PMC7872286; doi:10.1371/journal.pone.0246480)
Supplement: S1 File — (DOCX) [file pone.0246480.s001.docx]

**Supporting Information**

Comparative efficacy and safety of statin and fibrate monotherapy: A systematic review and meta-analysis of head-to-head randomized controlled trials

Table of Contents

[Literature search 2](#_Toc59542767)

[Exclusion criteria coding 6](#_Toc59542768)

[Risk of bias assessment 7](#_Toc59542769)

[Reporting bias assessment 9](#_Toc59542770)

[Summary of findings 13](#_Toc59542771)

[Subgroup analyses 15](#_Toc59542772)

# Literature search

**Ovid MEDLINE® and Epub Ahead of Print, In-Process & Other Non-Indexed Citations and Daily (1946 to 30 October 30 2019)**

1. exp fibric acid derivative/

2. exp Fibric Acids/ or fibrate$.tw. or fibric acid$.tw.

3. exp Gemfibrozil/ or gemfibro#il.tw.

4. exp bezafibrate/ or be#afibrate.tw.

5. exp clofibrate/ or exp clofibric acid/ or clofibr$.tw.

6. ciprofibrate.tw.

7. exp Fenofibrate/or procetofen.tw.

8. pemafibrate.tw.

9. (befibrat or befizal or beza or bezalip or bezacur or bezafibratum).tw.

10. (gemfibril or gemfibromax or gemhexal or gemizol or gemlipid).tw.

11. lopid.tw.

12. or/1-11

13. exp Hydroxymethylglutaryl-CoA Reductase Inhibitors/

14. hydroxymethylglutaryl$.tw.

15. HMG-CoA*.tw.

16. statin$.tw.

17. atorvastatin.tw.

18. cerivastatin.tw.

19. fluvastatin.tw.

20. lovastatin.tw.

21. pitavastatin.tw.

22. pravastatin.tw.

23. rosuvastatin.tw.

24. simvastatin.tw.

25. lipitor.tw.

26. baycol.tw.

27. lescol.tw.

28. mevacor.tw.

29. pravachol.tw.

30. zocor.tw.

31. lescol.tw.

32. liva*o.tw.

33. crestor.tw.

34. or/13-33

35. exp Cardiovascular Diseases/

36. exp Cerebrovascular Disorders/

37. exp Coronary disease/

38. exp Heart Failure/

39. exp HYPERLIPIDEMIAS/

40. (cardio$ or cardia$).tw.

41. cardio$ death.tw.

42. (heart$ or coronary$).tw.

43. angina*.tw.

44. revasculari*ation.tw.

45. (hyperlipid* or hypercholesterol*).tw.

46. (hyperlip?emia* or hypercholester?emia*).tw.

47. triglycerid*.tw.

48. hypertriglycerid?emia*.tw.

49. hyperlipoprotein?emia*.tw.

50. exp Lipoproteins, LDL/

51. exp Lipoproteins, HDL/

52. exp TRIGLYCERIDES/

53. LDL.tw.

54. HDL.tw.

55. exp Mortality/

56. mortalit$.tw.

57. exp Muscular Diseases/

58. exp renal failure/

59. or/35-58

60. 12 and 34 and 59

61. exp animals/ not humans.sh.

62. 60 not 61

63. randomized controlled trial.pt. or randomized.mp. or placebo.mp.

64. 62 and 63

**EMBASE (1974 to 30 October 2019)**

1. exp fibric acid derivative/

2. (fibrate$ or fibric acid$).tw.

3. exp gemfibrozil/ or gemfibro#il.tw.

4. exp bezafibrate/ or be#afibrate.tw.

5. exp pemafibrate/

6. exp clofibric acid/ or exp clofibrate derivative/ or exp clofibrate/ or etofylline clofibrate/ or exp clofibrate aluminum/ or clofibr$.tw.

7. exp ciprofibrate/ or ciprofibrate.tw.

8. exp choline fenofibrate/ or exp fenofibrate/ or exp fenofibric acid/ or procetofen.tw.

9. (befibrat or befizal or beza or bezalip or bezacur or bezafibratum).tw.

10. lopid.tw.

11. (gemfibril or gemfibromax or gemhexal or gemizol or gemlipid).tw.

12. or/1-11

13. exp hydroxymethylglutaryl coenzyme A reductase inhibitor/

14. hydroxymethylglutaryl$.tw.

15. HMG-CoA*.tw.

16. statin$.tw.

17. exp atorvastatin/ or atorvastatin.tw.

18. exp cerivastatin/ or cerivastatin.tw.

19. exp fluvastatin/ or fluvastatin.tw.

20. exp mevinolin/ or lovastatin.tw.

21. exp pitavastatin/ or pitavastatin.tw.

22. exp pravastatin/ or pravastatin.tw.

23. exp rosuvastatin/ or rosuvastatin.tw.

24. exp simvastatin/ or simvastatin.tw.

25. lipitor.tw.

26. baycol.tw.

27. lescol.tw.

28. mevacor.tw.

29. pravachol.tw.

30. zocor.tw.

31. liva*o.tw.

32. crestor.tw.

33. or/13-32

34. exp cardiovascular disease/

35. exp cerebrovascular disease/

36. exp coronary artery disease/

37. exp heart failure/

38. exp hyperlipidemia/

39. (cardio$ or cardia$).tw.

40. (heart$ or coronary$).tw.

41. angina*.tw.

42. revasculari*ation.tw.

43. (hyperlipid* or hypercholesterol*).tw.

44. (hyperlip?emia* or hypercholester?emia*).tw.

45. triglycerid*.tw.

46. hypertriglycerid?emia*.tw.

47. hyperlipoprotein?emia*.tw.

48. exp cholesterol blood level/ or exp cholesterol level/

49. exp low density lipoprotein cholesterol/ or exp low density lipoprotein/

40. exp high density lipoprotein cholesterol/ or exp high density lipoprotein/

51. exp triacylglycerol/

52. LDL.tw.

53. HDL.tw.

54. exp mortality/

55. mortalit$.tw.

56. exp musculoskeletal disease/

57. exp kidney injury/

58. or/34-57

59. 12 and 33 and 58

60. 59 not ((exp animal/ or nonhuman/) not exp human/)

61. random:.tw. or placebo:.mp. or double-blind:.tw.

62. 60 and 61

**Cochrane Library (inception to Issue 10 of 12, October 2019)**

Hydroxymethylglutaryl-CoA Reductase Inhibitors OR Statin OR atorvastatin OR rosuvastatin OR simvastatin OR fluvastatin OR pravastatin OR lovastatin OR pitavastatin in All Text AND Fibric Acids OR fibrate OR gemfibrozil OR fenofibrate OR bezafibrate OR pemafibrate OR clofibrate OR ciprofibrate in All Text

**ClinicalTrials.gov (on expert search page, inception to 30 October 2019)**

(Statin OR "Hydroxymethylglutaryl CoA Reductase Inhibitors" OR atorvastatin OR rosuvastatin OR simvastatin OR fluvastatin OR pravastatin OR lovastatin OR pitavastatin) AND ( fibrate OR "fibric acid" OR gemfibrozil OR fenofibrate OR bezafibrate OR pemafibrate) AND INFLECT EXACT "Interventional" [STUDY-TYPES]

**International Clinical Trials Registry Platform Search Portal (on advanced search page, inception to 30 October 2019)**

(Statin OR atorvastatin OR rosuvastatin OR simvastatin OR fluvastatin OR pravastatin OR lovastatin OR pitavastatin) AND (fibrate OR gemfibrozil OR fenofibrate OR bezafibrate OR pemafibrate) in the intervention (default search without synonyms, recruiting status “ALL”)

# Exclusion criteria coding

Each full-text article was assessed for the following exclusion criteria beginning at the start of the list (Code 1).

| **Table 1.** Study exclusion criteria coding and examples | | |
| --- | --- | --- |
| Code | Description | Examples |
| Code 1 | Wrong type of study | - Observational studies, such as cohort studies, case reports, or case-series - Not randomized - Not controlled |
| Code 2 | Wrong intervention | - Statins and fibrates were given in combination therapy with only statin or fibrate or placebo as comparator group; statins vs placebo; statins vs other statins; fibrates vs placebo; fibrates vs other fibrates; and no statin/fibrate for comparison - Conversion or switching to a statin or fibrate - Head-to-head comparison followed by combination therapy - Single treatment arm - Reported as nonstatin group/non-fibrate group as a whole (included other nonstatin medications) |
| Code 3 | Wrong outcomes | - Did not report any death or cardiovascular outcome of interest (CAD, MI, stroke, and angina) |
| Code 4 | Wrong population | - Participants <18 years old |
| Code 5 | Animal studies | - In-vivo study in animals or laboratory studies |
| Code 6 | Wrong study design for key questions | - Pharmacoeconomic studies - Pharmacokinetic studies (e.g., bioavailability) - Drug-drug/food interaction |
| Code 7 | Wrong study period | - Mean (or median) follow-up < 28 days |
| Code 8 | Using original studies instead | - The article is a systematic review (and meta-analysis) compiled of individual study data - Conference abstract with corresponding study with full publication identified - There is another report with outcome of interest is included (e.g. full study, clinical trial registration) |
| Code 9 | Unable to obtain full paper | - Only abstract available from database and full text not located |
| Code 10 | No results available | - Final result not yet published - Premature termination of study with no outcomes reported |

# Risk of bias assessment


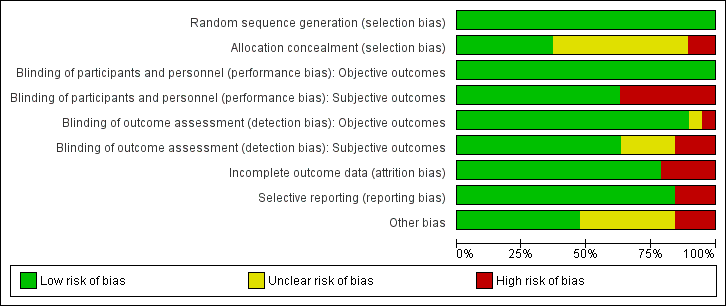


**Figure 1.** Risk of bias graph: review authors' judgements about each risk of bias item presented as percentages across all included studies


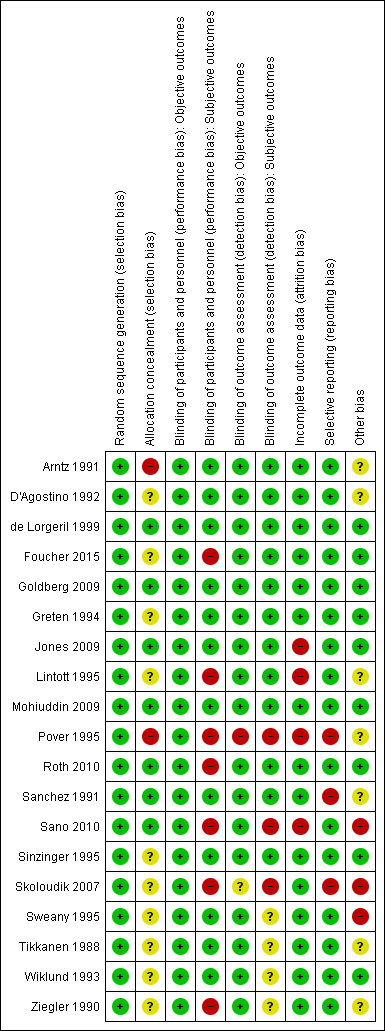


**Figure 2.** Risk of bias summary: review authors' judgements about each risk of bias item for each included study

# Reporting bias assessment


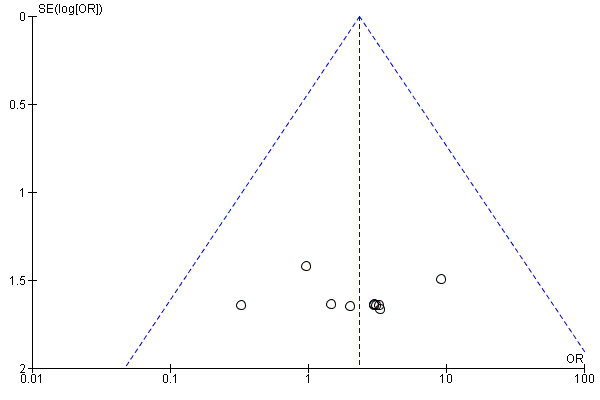
**Figure 3.** Funnel plot of comparison: statins versus fibrates, outcome: cardiovascular mortality


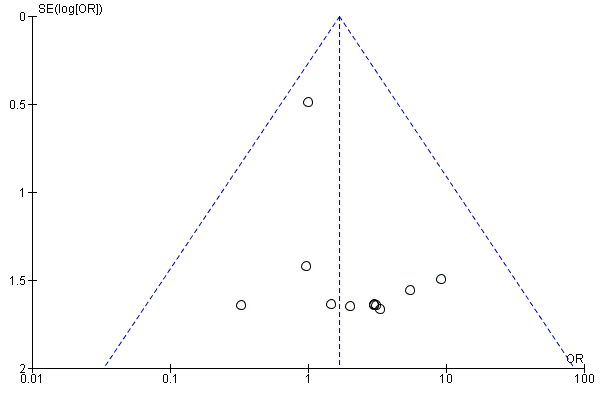
**Figure 4.** Funnel plot of comparison: statins versus fibrates, outcome: all-cause mortality


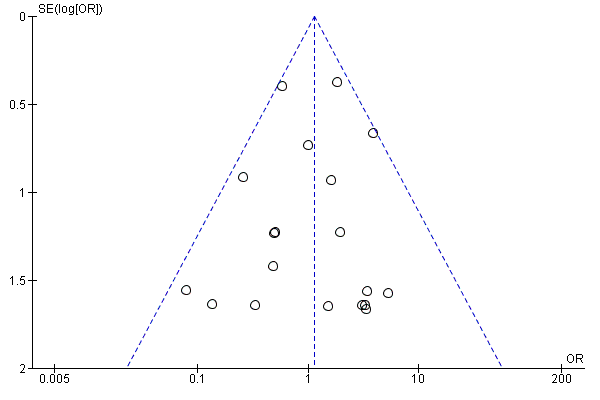
**Figure 5.** Funnel plot of comparison: statins versus fibrates, outcome: major cardiovascular events


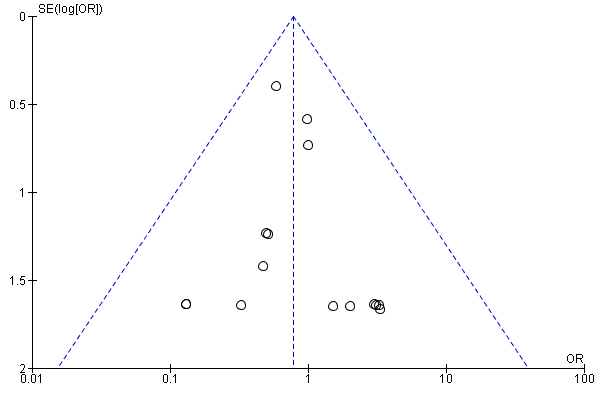
**Figure 6.** Funnel plot of comparison: statins versus fibrates, outcome: myocardial infarction


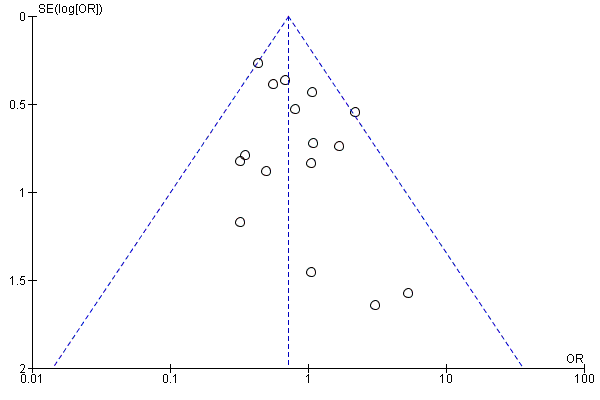
**Figure 7.** Funnel plot of comparison: statins versus fibrates, outcome: participant withdrawal due to adverse effects


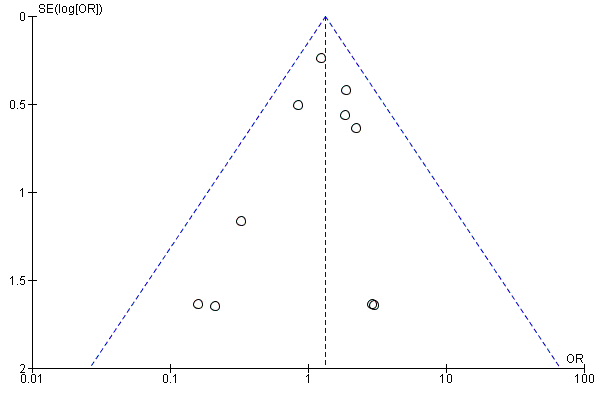
**Figure 8.** Funnel plot of comparison: statins versus fibrates, outcome: myalgia


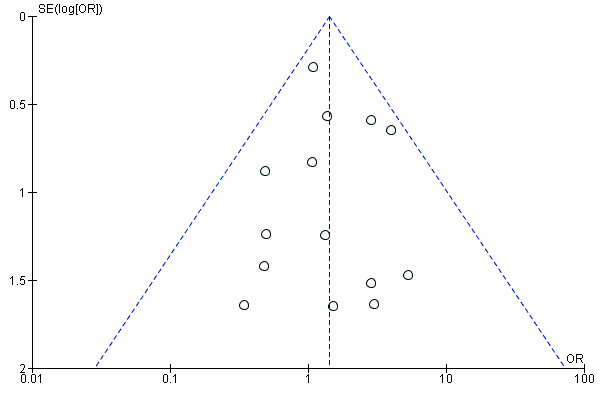
**Figure 9.** Funnel plot of comparison: statins versus fibrates, outcome: elevated creatine kinase

| Summary of findings | | | | | | |
| --- | --- | --- | --- | --- | --- | --- |
| **Statins compared to fibrates for adults with dyslipidemia** | | | | | | |
| **Patient or population**: adults with dyslipidemia  **Setting**: outpatients  **Intervention**: statins  **Comparison**: fibrates | | | | | | |
| Outcomes | **Anticipated absolute effects^*^** (95% CI) | | Relative effect (95% CI) | № of participants  (studies) | Certainty of the evidence (GRADE) | Comments |
|  | **Risk with fibrates** | **Risk with statins** |  |  |  |  |
| Cardiovascular Mortality follow up: range 10 weeks to 2 years | 2 per 1,000 | **4 per 1,000** (1 to 9) | **OR 2.35** (0.94 to 5.86) | 2657 (10 RCTs) | ⨁⨁◯◯ LOW ^a,b^ |  |
| All-cause mortality follow up: range 10 weeks to 2 years | 4 per 1,000 | **7 per 1,000** (3 to 13) | **OR 1.67** (0.87 to 3.22) | 5124 (11 RCTs) | ⨁⨁◯◯ LOW ^b,c^ |  |
| Major cardiovascular events follow up: range 10 weeks to 2 years | 16 per 1,000 | **18 per 1,000** (13 to 26) | **OR 1.15** (0.80 to 1.65) | 7619 (19 RCTs) | ⨁⨁◯◯ LOW ^b,c^ |  |
| Study withdrawal due to adverse effects follow up: range 10 weeks to 12 months | 57 per 1,000 | **41 per 1,000** (32 to 53) | **OR 0.71** (0.55 to 0.93) | 4680 (16 RCTs) | ⨁⨁◯◯ LOW ^d,e^ | 5 studies allowed statin dose to be increased, and one study allowed the treating clinician to select the starting dose of either statin or fibrate. |
| Serious adverse effects follow up: range 10 weeks to 24 weeks | 26 per 1,000 | **15 per 1,000** (10 to 24) | **OR 0.57** (0.36 to 0.91) | 3749 (9 RCTs) | ⨁⨁⨁◯ MODERATE ^c^ | 1 study allowed statin dose to be increased. |
| Myalgia follow up: range 12 weeks to 12 months | 24 per 1,000 | **31 per 1,000** (23 to 43) | **OR 1.32** (0.95 to 1.83) | 6090 (10 RCTs) | ⨁⨁◯◯ LOW ^b,c^ |  |
| Elevated serum alanine aminotransferase (ALT) follow up: range 12 weeks to 12 months | 29 per 1,000 | **41 per 1,000** (30 to 56) | **OR 1.43** (1.03 to 1.99) | 5225 (7 RCTs) | ⨁⨁◯◯ LOW ^b,f^ |  |
| Elevated serum creatinine follow up: range 10 weeks to 24 weeks | 35 per 1,000 | **6 per 1,000** (3 to 13) | **OR 0.17** (0.08 to 0.36) | 2553 (6 RCTs) | ⨁⨁⨁⨁ HIGH | 4 cases of kidney injury (reported as renal failure, renal impairment, or renal dysfunction, in 3 studies) occurred in the fibrate group and 0 in the statin group. |
| ***The risk in the intervention group** (and its 95% confidence interval) is based on the assumed risk in the comparison group and the **relative effect** of the intervention (and its 95% CI).   **CI:** Confidence interval; **OR:** Odds ratio; **MD:** Mean difference; **RR:** Risk ratio | | | | | | |
| **GRADE Working Group grades of evidence** **High certainty:** We are very confident that the true effect lies close to that of the estimate of the effect **Moderate certainty:** We are moderately confident in the effect estimate: The true effect is likely to be close to the estimate of the effect, but there is a possibility that it is substantially different **Low certainty:** Our confidence in the effect estimate is limited: The true effect may be substantially different from the estimate of the effect **Very low certainty:** We have very little confidence in the effect estimate: The true effect is likely to be substantially different from the estimate of effect | | | | | | |

#### Explanations

a. High risk or unclear risk of bias for allocation concealment, incomplete outcome data, blinding of outcome assessment, and selective reporting in several studies.

b. The estimate is imprecise because the 95% CI includes clinically relevant harm as well as clinically relevant benefit.

c. High risk or unclear risk of bias on several domains in several studies.

d. High risk of bias for blinding of participants and personnel and incomplete outcome data in several studies. High risk and unclear risk of other bias (see comment).

e. Funnel plot suggests reporting bias.

f. Surrogate outcome for liver toxicity.

# Subgroup analyses

Forest plots show the odds ratios (OR) and 95% confidence intervals (CI) of subgroups according to baseline study population characteristics: cardiovascular prevention status (primary, secondary, or not reported), type of dyslipidemia (primary hypercholesterolemia, mixed dyslipidemia, or other), and fibrate drug (bezafibrate, gemfibrozil, or fenofibrate).


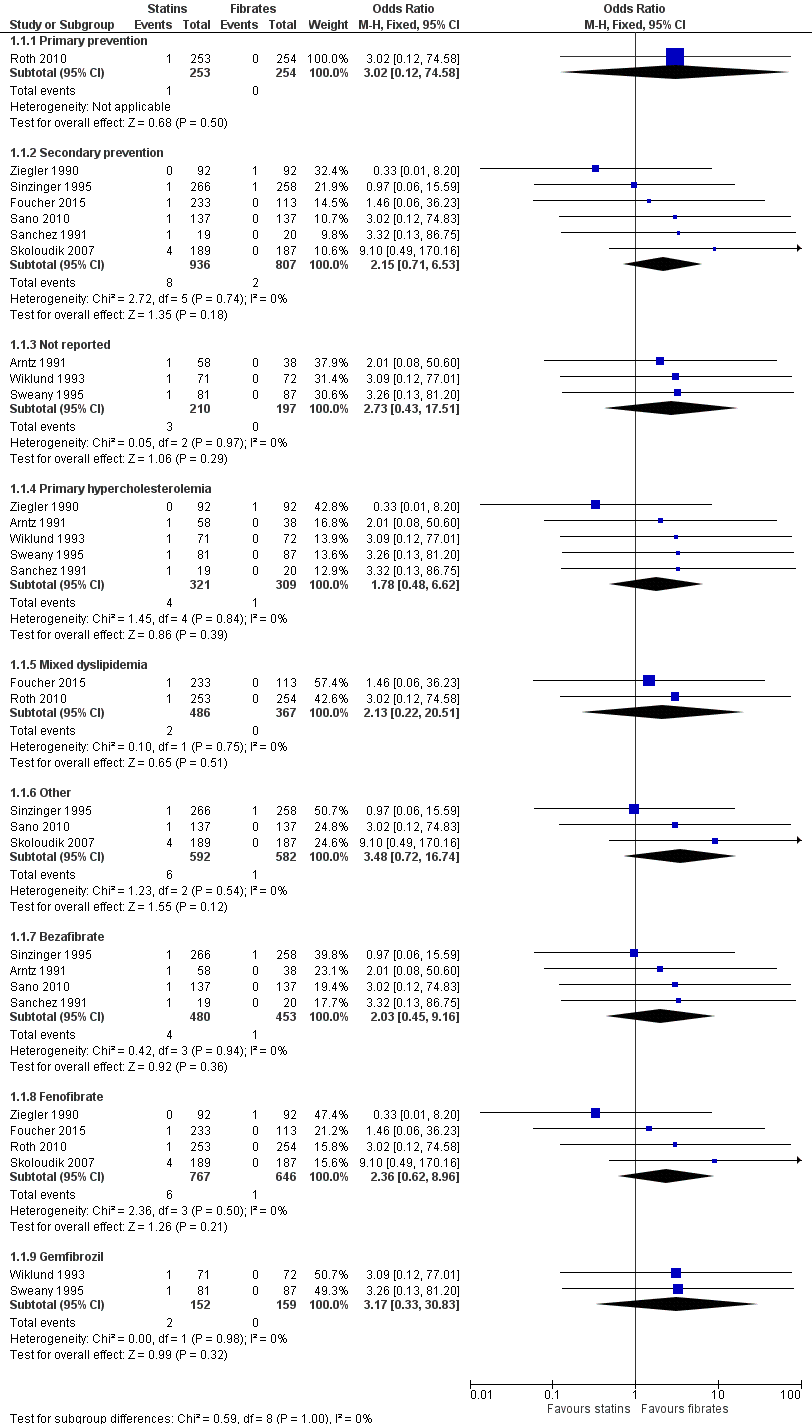
**Figure 10.** Forest plot of comparison: statins versus fibrates, outcome: cardiovascular mortality

**
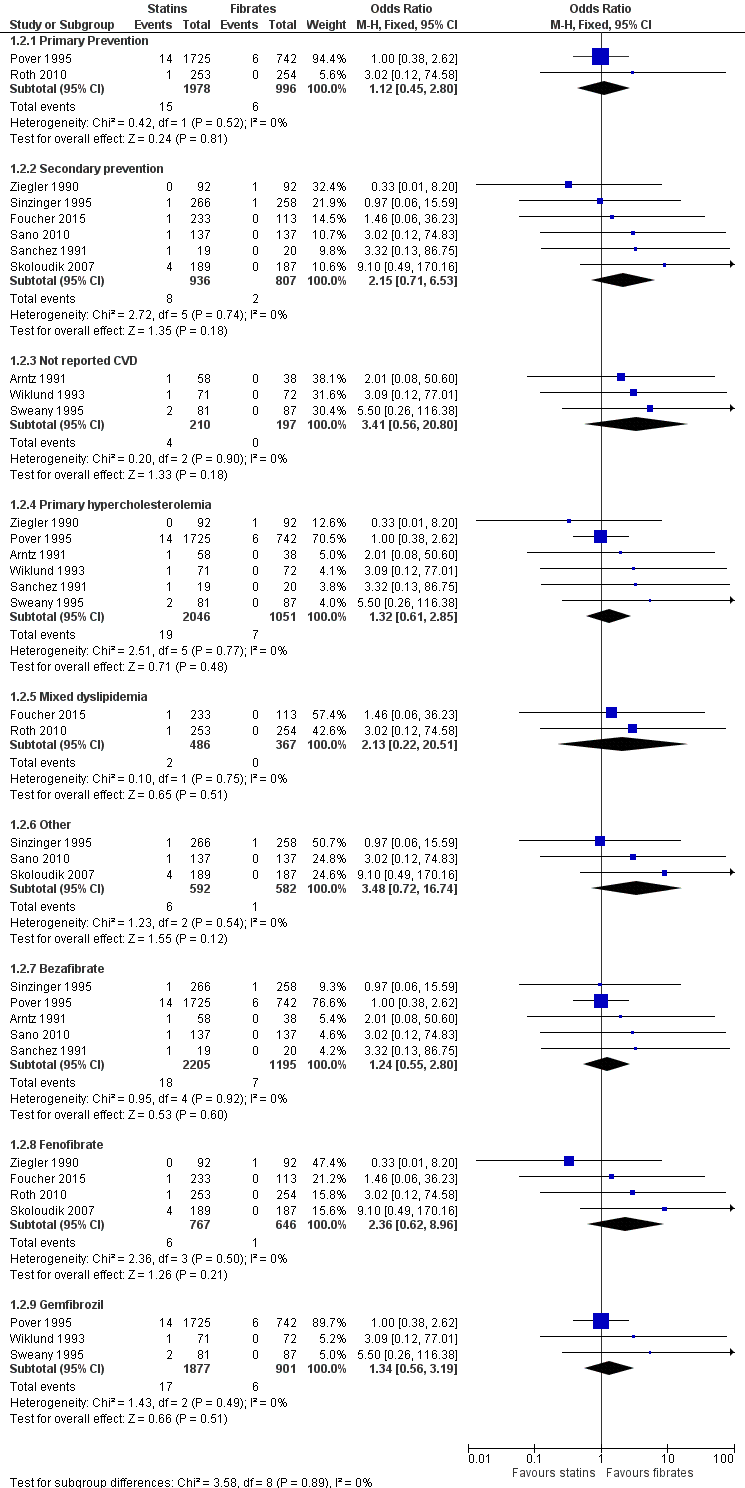
Figure 11.** Forest plot of comparison: statins versus fibrates, outcome: all-cause mortality

**
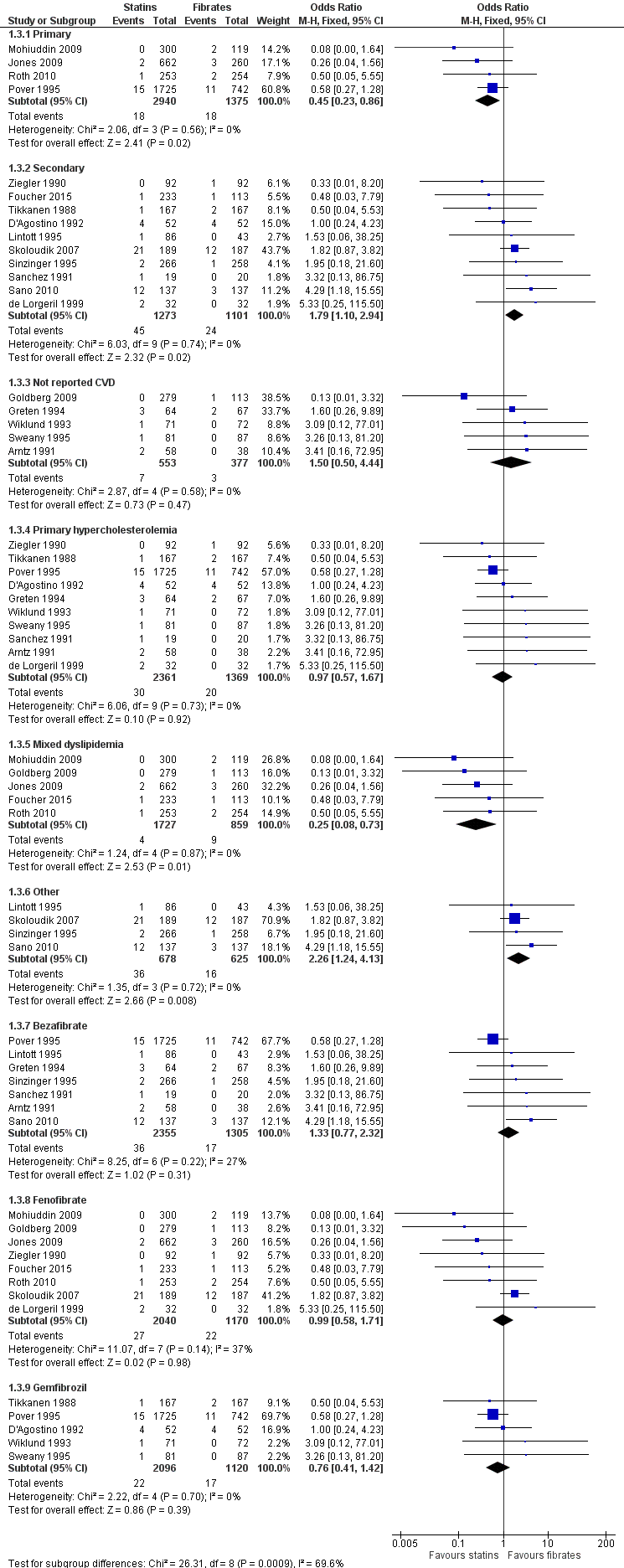
Figure 12.** Forest plot of comparison: statins versus fibrates, outcome: major cardiovascular events

**Figure 13.** Forest plot of comparison: statins versus fibrates, outcome:
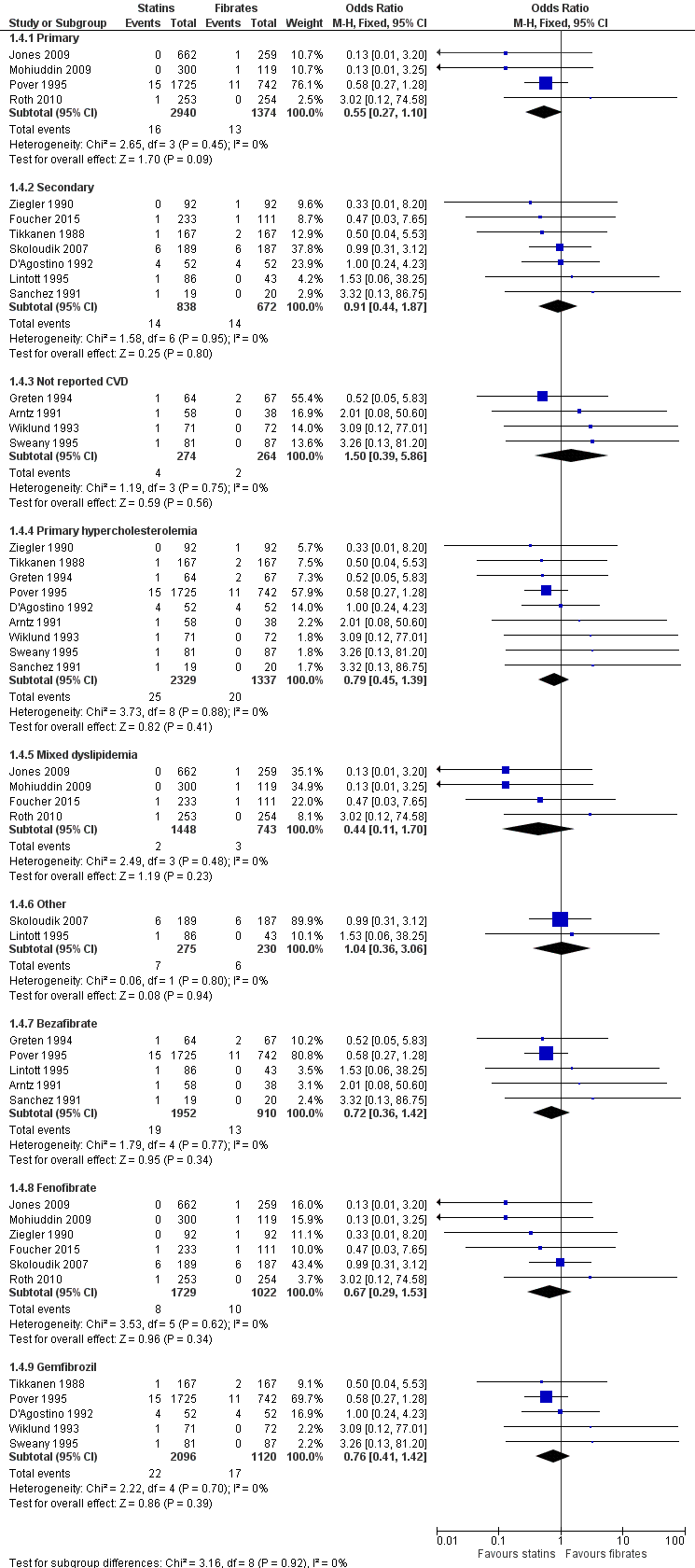
myocardial infarction

**Figure 14.** Forest plot of comparison: statins versus fibrates, outcome:
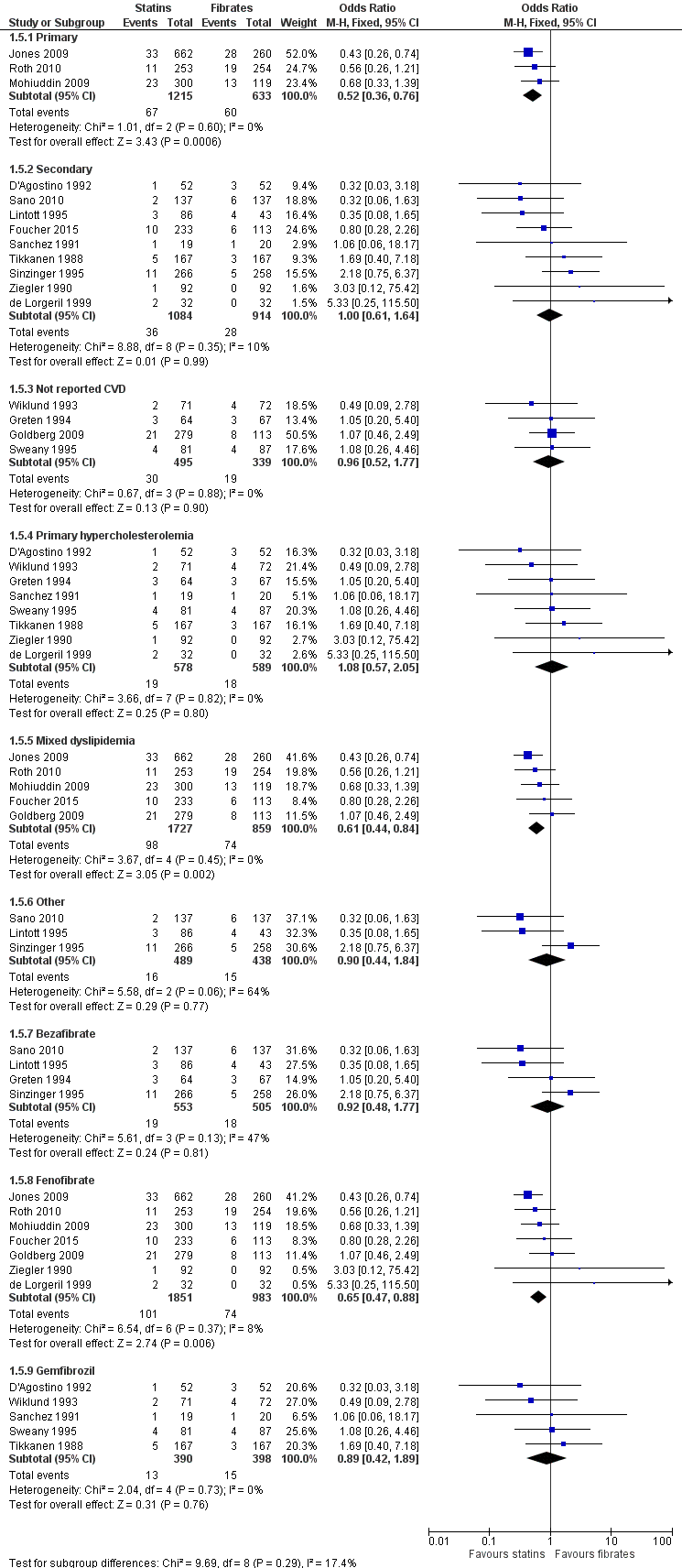
study withdrawal due to adverse effects

**Figure 15.** Forest plot of comparison: statins versus fibrates, outcome:
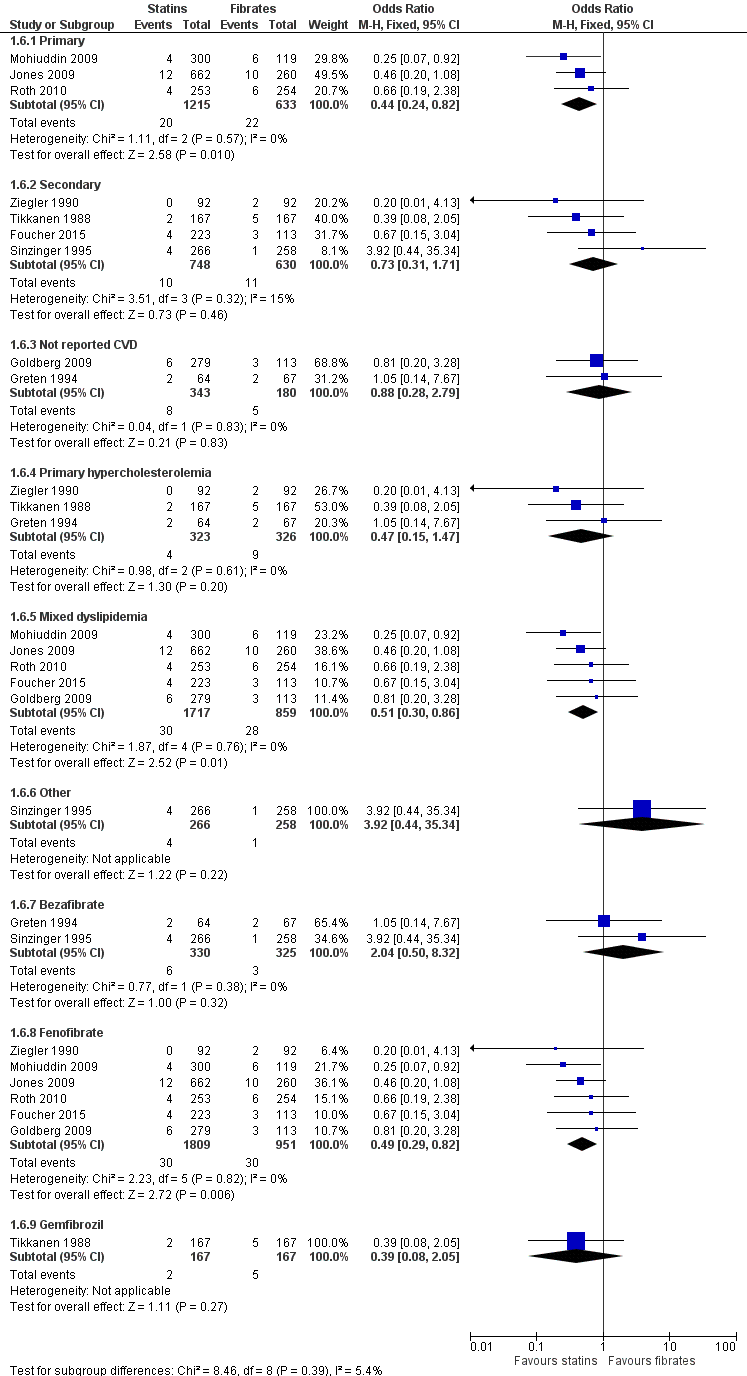
 serious adverse effects


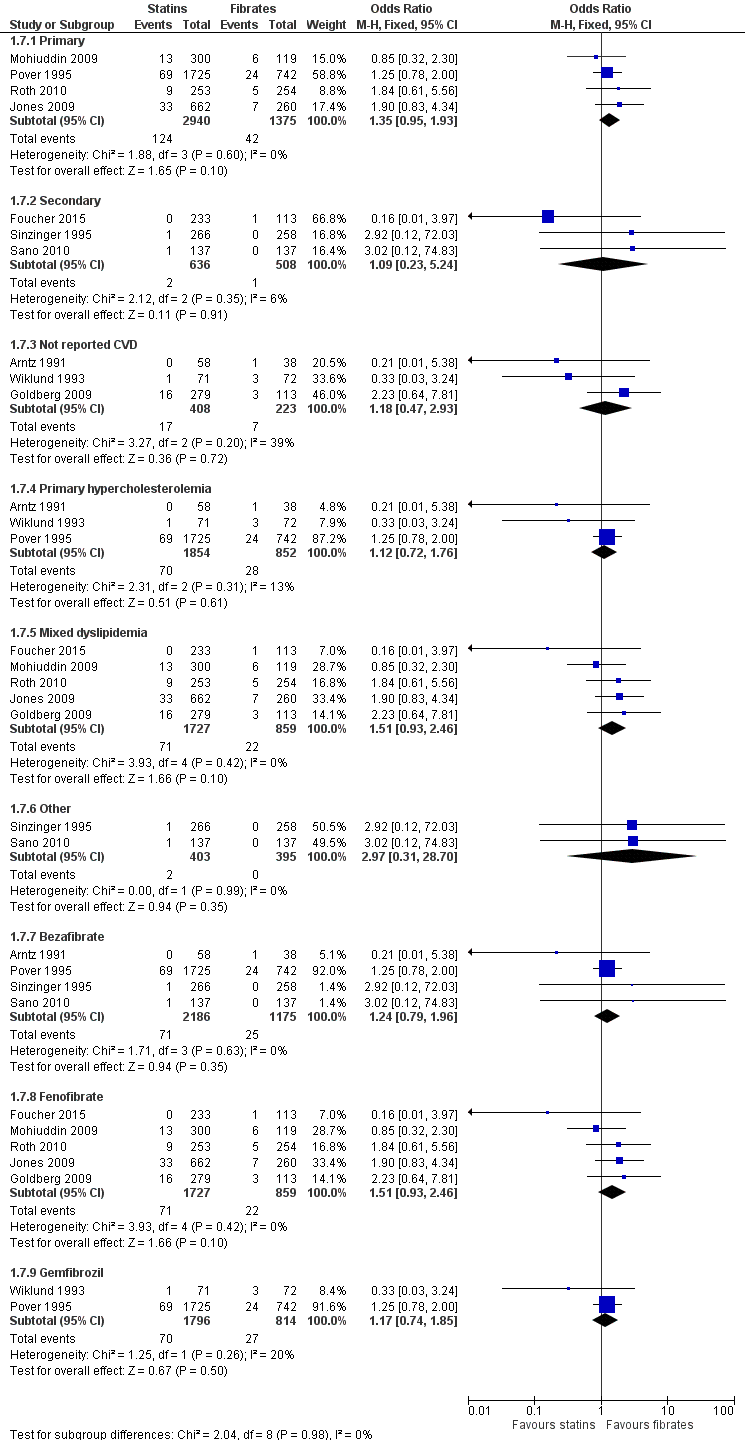
 **Figure 16.** Forest plot of comparison: statins versus fibrates, outcome: myalgia


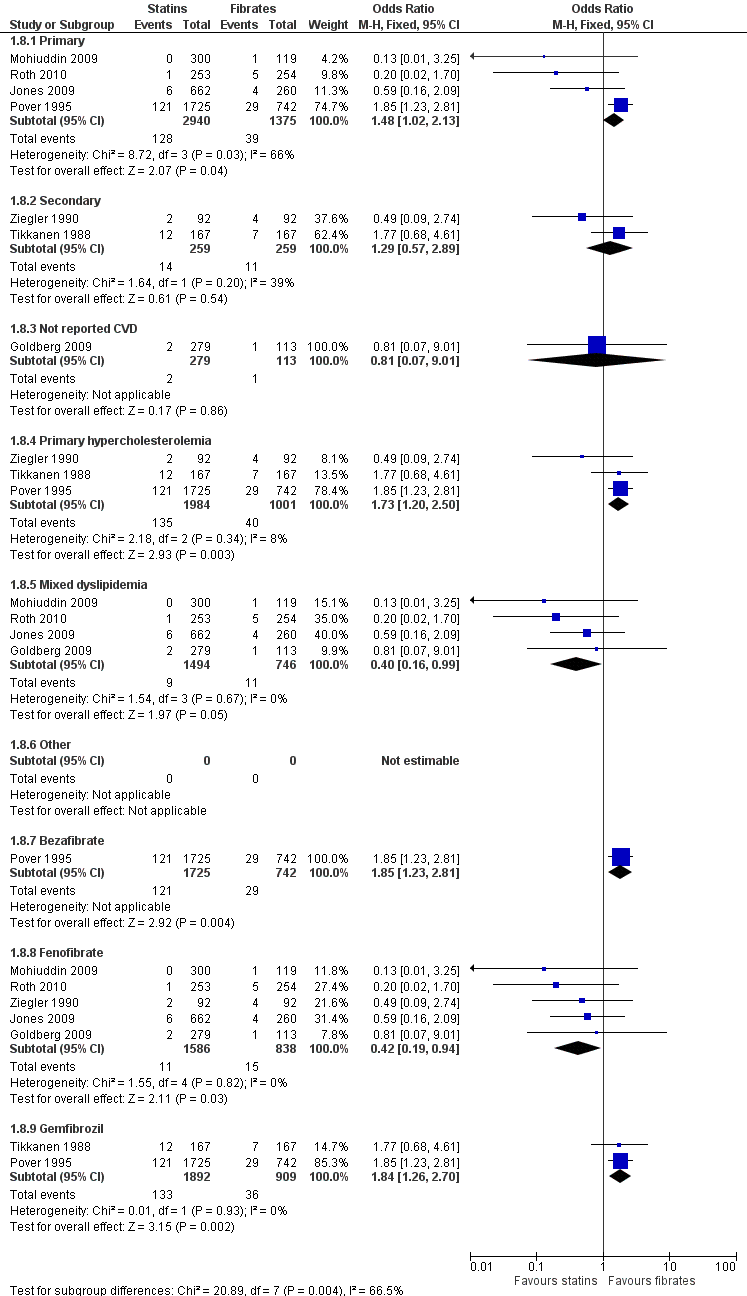
**Figure 17.** Forest plot of comparison: statins versus fibrates, outcome: elevated alanine aminotransferase

**
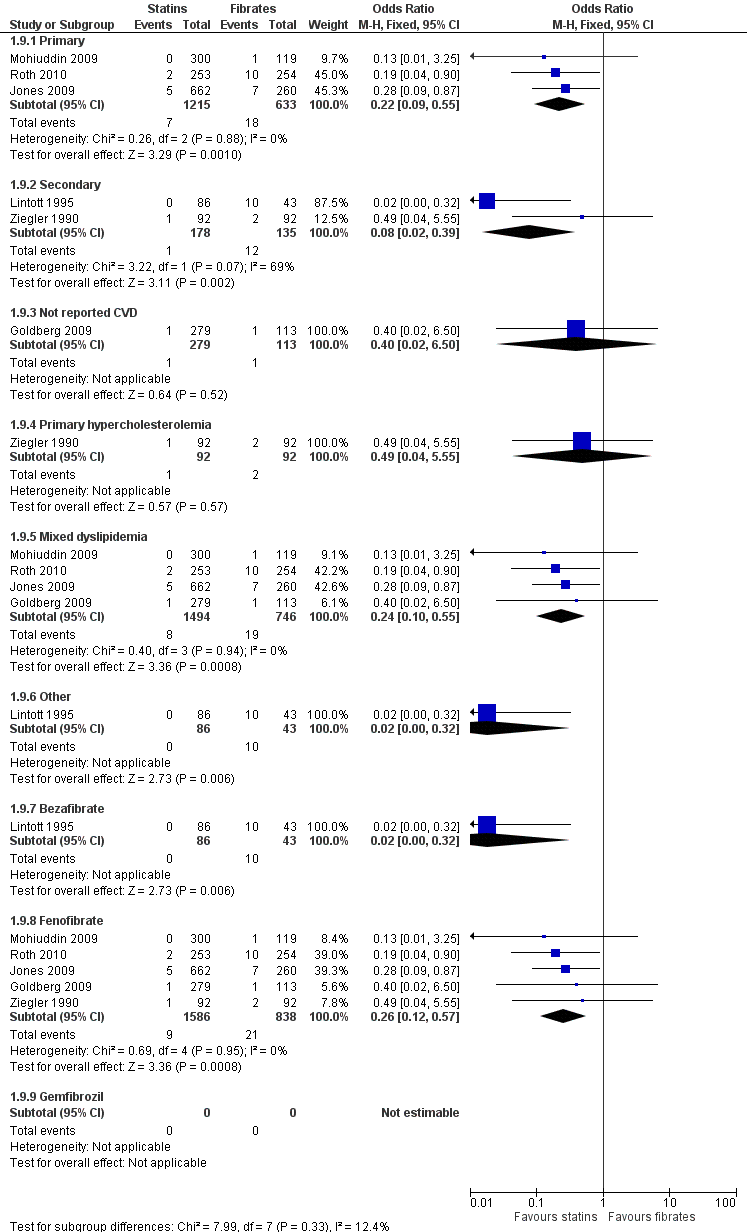
Figure 18.** Forest plot of comparison: statins versus fibrates, outcome: elevated serum creatinine
