# Supplementary material for: Comparative efficacy and safety of statin and fibrate monotherapy: A systematic review and meta-analysis of head-to-head randomized controlled trials
Source: PLoS One. 2021 Feb 9;16(2):e0246480. doi: 10.1371/journal.pone.0246480 (PMC7872286; doi:10.1371/journal.pone.0246480)
Supplement: S1 Protocol — (PDF) [file pone.0246480.s003.pdf]

Protocol Version: 1

Title: Comparative Efficacy and Safety of Statins and Fibrates in Prevention of Cardiovascular Events and Mortality: Protocol for a Systematic Review and Meta-analysis

Registration: This study is not submitted for registration.

Authors: Kin Y Tong[1], Joseph E Blais[2], Esther W Chan[2]

[1] Pfizer Upjohn Hong Kong Limited, Hong Kong Special Administrative Region, China, and formerly a student of the Department of Pharmacology and Pharmacy, Li Ka Shing Faculty of Medicine, University of Hong Kong, Hong Kong Special Administrative Region, China
[2] Centre for Safe Medication Practice and Research, Department of Pharmacology and Pharmacy, Li Ka Shing Faculty of Medicine, University of Hong Kong, Hong Kong Special Administrative Region, China

Contact Information

Ms Kin Y Tong
Department of Pharmacology and Pharmacy, Li Ka Shing Faculty of Medicine, University of Hong Kong, Hong Kong Special Administrative Region, China
Tel: (852) 9605 0300
Email: glortky@connect.hku.hk

Mr Joseph E Blais
Centre for Safe Medication Practice and Research, Department of Pharmacology and Pharmacy, Li Ka Shing Faculty of Medicine, University of Hong Kong, Hong Kong Special Administrative Region, China
Tel: (852) 2831 5119
Email: jblais@hku.hk

Dr Esther W Chan
Research Lead, Centre for Safe Medication Practice and Research
Department of Pharmacology and Pharmacy, Li Ka Shing Faculty of Medicine
The University of Hong Kong, Hong Kong Special Administrative Region, China
Office 02-08, 2/F Laboratory Block, 21 Sassoon Road, Hong Kong Special Administrative Region, China
Tel: (852) 2831 5110
Email: ewchan@hku.hk

Contributions:

- Search strategy development: JB, KT
- Procedures for selecting the study including screening: KT, JB
- Development of study eligibility, selection criteria, and data extraction criteria: KT, JB
- Drafting of the protocol: KT
- Read, revised and final approval of the protocol: All authors

- Guarantor: EC

Amendments:
In the event of protocol amendments, the date of each amendment, the change and the rationale will be described in this section. Changes will not be incorporated into the protocol body.

Funding: This study is not supported by any funding.

Conflict of interest: When this work began, Ms Tong was both a student at the University of Hong Kong and an employee at Otsuka Pharmaceutical (H.K.) Ltd. Effective August 19, 2019, she is employed at Pfizer Upjohn Hong Kong Limited. This study was conducted and led independently by the University of Hong Kong. Neither companies were involved in any aspect of this study. Dr. Chan reports receiving honorariums from the Hong Kong Hospital Authority, grants from Research Grants Council (Hong Kong), grants from Research Fund Secretariat of the Food and Health Bureau, grants from National Natural Science Fund of China, grants from Wellcome Trust, grants from Bayer, grants from Bristol-Myers Squibb, grants from Pfizer, grants from Janssen, grants from Amgen, grants from Takeda, grants from Narcotics Division of the Security Bureau of the Government of the Hong Kong SAR, unrelated to this study.

Introduction

Cardiovascular disease (CVD) remains one of the leading causes of morbidity and mortality worldwide (1). It includes coronary heart disease, cerebrovascular disease, rheumatic heart disease and other related conditions and in 2016 accounted for 17.9 million deaths, representing 31% of all deaths worldwide (2).

The prevention of CVD aims to eliminate events or minimize the impact of CVDs and their related disabilities by a coordinated set of actions, at population or individual level (1).

Among risks factors for CVD, hypercholesterolemia is considered to be a contributor to atherosclerosis. Over time, different lipid regulating drugs have been developed to prevent cardiovascular events. Among all the agents, the class of drugs known as statins, is most commonly prescribed. Comprehensive studies and reviews have demonstrated their effect in reducing all-cause mortality, combined fatal and non-fatal CVD and coronary heart disease (CHD), stroke, as well as revascularization rates (3, 4).

Fibrates, also known as fibric acid derivatives, are another common class of lipid regulating drug. The primary role for fibrates currently is in hypertriglyceridemia management (5). Despite a systematic review on primary prevention of CVD has reported fibrates' effect in combined CHD death and stroke (6), various studies and reviews have demonstrated that fibrates, compared to placebo treatment, show only effect on non-fatal coronary

events, such as myocardial infarction, but not on all-caused or cardiovascular mortality, or stroke events (7-10).

Despite statins' proven clinical benefits compared with placebo, statins may not be the drug of choice for all patients, given their association with muscle injury, rare instances of hepatotoxicity and new-onset type 2 diabetes (11). Many patients may be at increased risk of these side effects or may have experienced side effects, and thus would prefer a non-statin treatment alternative. In regards to the efficacy demonstrated in clinical trials, fibrates are a potential alternative drug for patients who cannot tolerate statins, patients with or at risk of type 2 diabetes or patients with dyslipidemia, due to the well-established safety profile of fibrates (7, 12).

Different studies using a variety of pharmacological agents for prevention of CVD exist. However, indexes or measurements as an indicator or risk of CVD or outcomes vary to a large extent. In addition, comparison among different classes of drugs is rather limited. To our knowledge, no previous systematic review has assessed the direct head-to-head efficacy and safety of statins as compared with fibrates in terms of reducing clinically meaningful cardiovascular events. As it is of clinical importance in comparing different strategies in management and prevention of cardiovascular diseases, we aim to evaluate how statins compare to fibrates in reducing the risk of cardiovascular events and in safety profile.

OBJECTIVE

We aim to directly assess, evaluate and compare statins and fibrates in terms of efficacy in reducing the risk of cardiovascular events and mortality.

Objective 1: What is the effect of statin monotherapy compared with fibrate monotherapy in improving cardiovascular mortality in adult patients, in the setting of diabetes diagnosis and for primary and secondary prevention of cardiovascular events.
Objective 2: What proportion of adult patients tolerate treatment and experience adverse effects with statin monotherapy as compared with fibrate monotherapy?

METHODS

Study design: This study is a systematic review with planned meta-analysis of published and unpublished work. The protocol is written in accordance with the PRISMA Statement for the conduct of meta-analyses of intervention studies (13).

Types of studies to be included: Randomized controlled trials comparing fibrates to statins.

Participants/ Population: Study population would include general population of adults (≥18 years old). All participants assigned to statin or fibrate monotherapy will be included.

Intervention and Comparator: Intervention and comparator arm will comprise of statin only and fibrate only treatment (monotherapy).

<u>Eligibility</u>

Inclusion criteria

- ❏ Participants aged ≥18 years
- ❏ Randomized controlled trials
- ❏ Directly assess the effect of statins compared with fibrates, and report at least a clinical cardiovascular outcome of interest or death
- ❏ Any pharmacological agents under the class of statins and fibrates
- ❏ Statin or fibrate therapy compared directly in a head-to-head fashion
- ❏ Minimum duration of mean (or median) follow-up of 28 days
- ❏ No language restriction

Exclusion criteria

- ❏ Participants aged <18 years
- ❏ Comparator group for statin or fibrates was given as combination therapy with another lipid modifying drug. For studies with multiple treatment arms, which may include combination treatment with a statin or fibrate or placebo, if separate statin and fibrate monotherapy data is available then the relevant data from the treatment arms will be included
- ❏ Do not have clinical outcome assessed
- ❏ Observational studies, including but not limited to cohort studies, case reports or case-series
- ❏ Animal studies

Information Sources

Literature search strategies will be developed with medical subject headings (MeSH) and text words related to cardiovascular diseases. We will search three electronic databases:

- ❏ Medline via Ovid interface (1946 onward),
- ❏ Embase via Ovid interface (1974 onward), and
- ❏ Cochrane Library (Cochrane Central Register of Controlled Trials) via Wiley Library.

PubMed will be searched for recent publication that have yet to be included in Medline.

Published literature will be supplemented by a grey literature search to limit the potential for publication bias. We will search for unpublished trials, ongoing studies, and study protocols using the following three sources:

- ❑ ClinicalTrials.gov,
- ❑ International Clinical Trials Registry Platform Search Portal (http://apps.who.int/trialsearch/), and
- ❑ Hand search of relevant study reference lists.

Databases will be searched using keywords and/or medical subject headings. MeSH will be used for MEDLINE, Cochrane Library, and PubMed, while EMTREE will be used for Embase. Advice from medical librarian will be sought for specific search strategies. Draft search strategies are included in Appendices. Finalized search strategy would be recorded. Duplicated references will be removed using Endnote.

Study Selection and Record

Two authors (KT, JB) will independently perform the search and screen study titles and abstracts identified in the search, using the listed inclusion and exclusion criteria. In case of discrepancy between the reviewers, the full reports will be obtained for assessment and resolved by consensus. Search results including the reasons for exclusion would be recorded.

Full text of potential studies for inclusion will be retrieved after the screening of title and abstract. All citations of identified articles will be managed with EndNote X9. Two authors (KT, JB) will cross-checked each study record for inclusion. If the two authors cannot agree on study inclusion, then a third study author (EC) will be contacted to assist with resolution.

Data Extraction

PICO items including, but not limited to, the data source of study, type of study design, demographics of participants (average age, gender, cardiovascular prevention status), intervention (statins and fibrates used, dosage, frequency and duration of treatment), outcomes of interest, will be recorded on a structured data extraction form. One reviewer (KT) will extract data independently and a second (JB) will independently check the data extraction. Disagreements will be resolved by discussion.

Outcomes

We will analyze and grade important clinical endpoints of interest to patients and doctors.

The primary efficacy outcome is defined as:

- ❑ **Cardiovascular mortality**: any death due to a confirmed or suspected cardiovascular cause.

Secondary efficacy outcomes are defined as:

- **All-cause mortality**: defined as a death due to any cause.
- **Major cardiovascular events**: defined as the number of participants in each study reported to experience cardiovascular mortality, coronary artery disease, myocardial infarction, unstable angina, or stroke (inclusive of cerebrovascular events and cerebral infarction, but not transient ischemic attack).
- **Coronary artery disease** includes reports of coronary artery disease and coronary events that are not specified otherwise as myocardial infarction or unstable angina.
- **Myocardial infarction**: defined as any report of fatal or non-fatal myocardial infarction.
- **Stroke**: defined as any report of fatal and non-fatal stroke, cerebrovascular event, or cerebral infarction.
- **Unstable angina**: defined as any occurrence reported as unstable angina (pectoris).

Secondary safety outcomes of interest include:

- **Medication tolerance**, which will be assessed by the **number of participants who withdraw from the study due to adverse effects** and the **number of serious adverse events** reported in each study.
- **Non-cardiovascular mortality**: defined as a death from any non-cardiovascular cause.
- **Liver function abnormality** will be assessed by elevation in alanine aminotransferase (ALT), which is defined as the number of participants who experience an elevated ALT (or elevated transaminase if no differentiation between ALT and AST) at the lowest (most conservative) threshold reported within each study. For example, if both elevations of ALT > 3 times the upper limit of normal (ULN) and > 5 times ULN are reported for the same study, then we will extract the number of subjects reported as having an ALT > 3 times ULN.
- **Muscle adverse events** will be assessed by the outcomes of **elevated creatine kinase** (CK) levels and occurrence of **myalgia**. Elevation in CK levels is defined as the definition within each study. If a study reports multiple thresholds for elevated CK, then the lowest (most conservative) threshold within each study will be used. For the outcome of myalgia, in each study we will first document the number of participants who report myalgia (muscle pain). If myalgia is not reported, then we will extract the number of participants reported as having musculoskeletal pain.
- **New diagnosis of diabetes** will be defined as a new diagnosis of diabetes mellitus following treatment assignment.
- **Renal adverse effects** will be assessed by the outcomes of **elevated serum creatinine** and the occurrence of **acute kidney injury**. Elevation in serum creatinine levels is defined as the definition within each study. If a study reports multiple thresholds, then the lowest (most conservative) threshold will be used.

In case some of the outcomes are reported as a composite measure, all composite outcomes will also be extracted together with individual outcomes as reported in the studies.

Quality assessment

To assess possible risk of bias for studies collected, information will be collected via Cochrane Collaboration tool for assessing the risk of bias, which is in Table 8.5.a in the Cochrane Handbook for Systematic Reviews of Interventions (14).

The below domains/criteria will be evaluated as a score in risk of bias:

- ❏ *Selection bias*: sequence generation and allocation concealment
- ❏ *Performance bias*: blinding of participants and personnel
- ❏ *Detection bias*: blinding of outcome assessment
- ❏ *Attrition bias*: incomplete outcome data
- ❏ *Reporting bias*: selective outcome reporting

Procedures undertaken or omitted in each study will be described. For the extracted information, the study will be rated in a scale of "high risk", "moderate risk" and "low risk". Graphic representation of potential bias within individual study and across studies will be presented by a funnel plot if more than 10 studies are available using RevMan 5.1 (Review Manager 5.1). Each domain in the risk of bias assessment will be considered independently without collation and assignment of an overall score.

Besides, quality components will be evaluated e.g. full-text publication versus abstracts, preliminary versus mature results, and published versus unpublished data

Assessment will be reported. Studies of any level of risk of bias will be included in the main analyses.

Qualitative Analysis

A systematic narrative synthesis will be provided. Information will be presented in text and tables as a summary and explanation of the designs and findings of the studies included in the synthesis. We will explore the association and findings in individual studies and between the included studies.

Studies of any level of risk of bias will be included in the analyses.

Meta-Analysis

If studies are sufficiently homogeneous in terms of design and comparator, meta-analysis will be conducted using a random effects model. Each outcome will be combined and calculated with RevMan 5.1 for statistical measurement, according to the statistical guidelines referenced in the current version (5.1.0) of the Cochrane Handbook for Systematic Reviews of Interventions.

Clinical heterogeneity will be considered with the variability in participant factors and trial factors, with reference to the PICO criteria stated above. Participant factors include the demographics of participants, while example of

trial factors include the intervention, duration of treatment, and allocation concealment.

Data Synthesis

All outcomes will be considered dichotomous. We will pool studies to estimate a risk ratio (RR) and 95% confidence interval (CI), using a random effects model.

Assessment of Heterogeneity

Statistical heterogeneity will be tested using I^2 statistic and the Chi^2 test. If tests of heterogeneity are significant (I^2>=50% or P<0.1), the study design and characteristics of the included studies will be analyzed by subgroup analysis or sensitivity analysis to address the source of heterogeneity.

Subgroup analysis will be based on the demographics of patients, types of treatments and level of prevention (primary or secondary) while sensitivity analysis will be based on quality components and risk of bias.

Missing Data

In incidence of missing data, we will attempt to contact the authors of the original study.

Subgroup and Sensitivity Analyses

Subgroup analyses will be conducted to compare the following:

- ❏ **CVD prevention population**: Studies for primary or secondary prevention of cardiovascular disease (CVD), with primary prevention referring to trials with both study groups having a baseline prevalence of CVD less than or equal to 10%. Secondary prevention will refer to studies with baseline prevalence of CVD > 10%. If a study does not report baseline history of CVD, then it will be classified as not reported.
- ❏ **Type of fibrate intervention**: we will group studies according to the specific fibrate treatment used in the study.
- ❏ **Type of dyslipidemia**: we will group studies based on the major type of dyslipidemia (e.g., primary hypercholesterolemia, mixed dyslipidemia, other) under study.
- ❏ **Studies of participants with and without diabetes**: Studies which had diabetes as inclusion criteria at baseline (>90% of patients with baseline diabetes), will be compared to those without diabetes.

To assess the robustness of our findings, sensitivity analyses will include removing the single highest weighted study for each outcome, and to remove any studies judged to be at high risk on any of the following three key domains: allocation sequence concealment, blinding of participants and personnel, and blinding of outcome assessment.

Certainty of Evidence Assessment

The summarized outcomes will be evaluated in terms of evidence quality with the Grading of Recommendations Assessment, Development and Evaluation (GRADE) working group methodology. Confidence in the cumulative estimate will be assessed across these domains: risk of bias across studies, consistency, precision, directness, and publication bias. Quality of evidence will be classified in a scale of “high”, “moderate”, “low” and “very low”.

Dissemination

This review will form the major part of the master’s dissertation for KT. The authors aim to publish the review in a high-quality general medical or cardiology journal.

References

1. Piepoli MF, Hoes AW, Agewall S, Albus C, Carlos Brotons, Catapano AL, et al. European Guidelines on cardiovascular disease prevention in clinical practice. *Eur Heart J*. 2016; **37**(29): 2315-81.
2. The World Health Organization. Cardiovascular diseases (CVDs). 2018. https://www.who.int/cardiovascular_diseases/en/ (accessed October 19, 2018)
3. Taylor F, Huffman M, Macedo A, Moore T, Burke M, Smith GD, et al. Statins for the primary prevention of cardiovascular disease. Cochrane Database of Systematic Reviews. 2013;(1):CD004816.
4. Studer M, Briel M, Leimenstoll B, Glass TR, Bucher HC. Effect of different antilipidemic agents and diets on mortality a systematic review. *Arch Intern Med*. 2015; **165**: 725-30.
5. Wiggins B, Saseen J, Morris P. Gemfibrozil in combination with statins - is it really contraindicated? *Curr Atheroscler Rep.* 2016; **18**(4): 18.
6. Jakob T, Nordmann AJ, Schandelmaier S, Ferreira-González I, Briel M. Fibrates for primary prevention of cardiovascular disease events. Cochrane Database of Systematic Reviews. 2016;(11):CD009753.s.
7. Saha SA, Arora RR. Fibrates in the prevention of cardiovascular disease in patients with type 2 diabetes mellitus - a pooled meta-analysis of randomized placebo-controlled clinical trials. *Int J Cardiol.* 2010; **141**(2): 157-66.
8. Saha SA, Kizhakepunnur LG, Bahekar A, Arora RR. The role of fibrates in the prevention of cardiovascular disease--a pooled meta-analysis of long-term randomized placebo-controlled clinical trials. *Am Heart J.* 2007; **154**(5): 943-53.
9. Abourbih S, Filion KB, Joseph L, Schiffrin EL, Rinfret S, Poirier P, et al. Effect of fibrates on lipid profiles and cardiovascular outcomes: a systematic review. *Am J Med.* 2009; **122**(10): 962 e1-8.
10. Jun M, Foote C, Lv J, Neal B, Patel A, Nicholls SJ, et al. Effects of fibrates on cardiovascular outcomes: a systematic review and meta-analysis. *Lancet.* 2010; **375**: 1875-84.
11. American College of Cardiology. Summarizing the current state and evidence on efficacy and safety of statin therapy. American College of Cardiology. 2016. https://www.acc.org/latest-in-cardiology/articles/2016/11/17/09/03/summarizing-the-current-state-and-evidence-on-efficacy-and-safety-of-statin-therapy. (accessed October 19, 2018)
12. Simental-Mendía L, Simental-Mendía M, Sánchez-García, Banach M, Atkin S, AM AJG, et al. Effect of fibrates on glycemic parameters: a systematic review and meta-analysis of randomized placebo-controlled trials. *Pharmacol Res.* 2018; **132**: 232-41.
13. Shamseer L, Moher D, Clarke M, Ghersi D, Liberati A, Petticrew M, et al. Preferred reporting items for systematic review and meta-analysis protocols (PRISMA-P) 2015: elaboration and explanation. *BMJ.* 2015; **349**: g7647.
14. Higgins J, Green S. Cochrane handbook for systematic reviews of interventions. Higgins J, Green S, editors. England: John Wiley & Sons Ltd.; 2008. 187-242.

Appendices

Appendix 1. Draft MEDLINE search - Ovid interface

1. exp fibric acid derivative/
2. exp Fibric Acids/ or fibrate$.tw. or fibric acid$.tw.
3. exp Gemfibrozil/ or gemfibro#il.tw.
4. exp bezafibrate/ or be#afibrate.tw.
5. exp clofibrate/ or exp clofibric acid/ or clofibr$.tw.
6. ciprofibrate.tw.
7. exp Fenofibrate/ or procetofen.tw.
8. pemafibrate.tw.
9. (befibrat or befizal or beza or bezalip or bezacur or bezafibratum).tw.
10. (gemfibril or gemfibromax or gemhexal or gemizol or gemlipid).tw.
11. lopid.tw.
12. or/1-11
13. exp Hydroxymethylglutaryl-CoA Reductase Inhibitors/
14. hydroxymethylglutaryl$.tw.
15. HMG-CoA*.tw.
16. statin$.tw.
17. atorvastatin.tw.
18. cerivastatin.tw.
19. fluvastatin.tw.
20. lovastatin.tw.
21. pitavastatin.tw.
22. pravastatin.tw.
23. rosuvastatin.tw.
24. simvastatin.tw.
25. lipitor.tw.
26. baycol.tw.
27. lescol.tw.
28. mevacor.tw.
29. pravachol.tw.
30. zocor.tw.
31. lescol.tw.
32. liva*o.tw.
33. crestor.tw.
34. or/13-33
35. exp Cardiovascular Diseases/
36. exp Cerebrovascular Disorders/
37. exp Coronary disease/
38. exp Heart Failure/
39. exp HYPERLIPIDEMIAS/
40. (cardio$ or cardia$).tw.
41. cardio$ death.tw.
42. (heart$ or coronary$).tw.
43. angina*.tw.
44. revasculari*ation.tw.
45. (hyperlipid* or hypercholesterol*).tw.
46. (hyperlip?emia* or hypercholester?emia*).tw.
47. triglycerid*.tw.

48. hypertriglycerid?emia*.tw.
49. hyperlipoprotein?emia*.tw.
50. exp Lipoproteins, LDL/
51. exp Lipoproteins, HDL/
52. exp TRIGLYCERIDES/
53. LDL.tw.
54. HDL.tw.
55. exp Mortality/
56. mortalit$.tw.
57. exp Muscular Diseases/
58. exp renal failure/
59. or/35-58
60. 12 and 34 and 59
61. exp animals/ not humans.sh.
62. 60 not 61
63. randomized controlled trial.pt. or randomized.mp. or placebo.mp.
64. 62 and 63

Appendix 2. Draft EMBASE search - Ovid interface

1. exp fibric acid derivative/
2. (fibrate$ or fibric acid$).tw.
3. exp gemfibrozil/ or gemfibro#il.tw.
4. exp bezafibrate/ or be#afibrate.tw.
5. exp pemafibrate/
6. exp clofibric acid/ or exp clofibrate derivative/ or exp clofibrate/ or etofylline clofibrate/ or exp clofibrate aluminum/ or clofibr$.tw.
7. exp ciprofibrate/ or ciprofibrate.tw.
8. exp choline fenofibrate/ or exp fenofibrate/ or exp fenofibric acid/ or procetofen.tw.
9. (befibrat or befizal or beza or bezalip or bezacur or bezafibratum).tw.
10. lopid.tw.
11. (gemfibril or gemfibromax or gemhexal or gemizol or gemlipid).tw.
12. or/1-11
13. exp hydroxymethylglutaryl coenzyme A reductase inhibitor/
14. hydroxymethylglutaryl$.tw.
15. HMG-CoA*.tw.
16. statin$.tw.
17. exp atorvastatin/ or atorvastatin.tw.
18. exp cerivastatin/ or cerivastatin.tw.
19. exp fluvastatin/ or fluvastatin.tw.
20. exp mevinolin/ or lovastatin.tw.
21. exp pitavastatin/ or pitavastatin.tw.
22. exp pravastatin/ or pravastatin.tw.
23. exp rosuvastatin/ or rosuvastatin.tw.
24. exp simvastatin/ or simvastatin.tw.
25. lipitor.tw.
26. baycol.tw.
27. lescol.tw.
28. mevacor.tw.
29. pravachol.tw.
30. zocor.tw.
31. liva*o.tw.
32. crestor.tw.
33. or/13-32
34. exp cardiovascular disease/
35. exp cerebrovascular disease/
36. exp coronary artery disease/
37. exp heart failure/
38. exp hyperlipidemia/
39. (cardio$ or cardia$).tw.
40. (heart$ or coronary$).tw.
41. angina*.tw.
42. revasculari*ation.tw.
43. (hyperlipid* or hypercholesterol*).tw.
44. (hyperlip?emia* or hypercholester?emia*).tw.
45. triglycerid*.tw.
46. hypertriglycerid?emia*.tw.

47. hyperlipoprotein?emia*.tw.
48. exp cholesterol blood level/ or exp cholesterol level/
49. exp low density lipoprotein cholesterol/ or exp low density lipoprotein/
50. exp high density lipoprotein cholesterol/ or exp high density lipoprotein/
51. exp triacylglycerol/
52. LDL.tw.
53. HDL.tw.
54. exp mortality/
55. mortalit$.tw.
56. exp musculoskeletal disease/
57. exp kidney injury/
58. or/34-57
59. 12 and 33 and 58
60. 59 not ((exp animal/ or nonhuman/) not exp human/)
61. random:.tw. or placebo:.mp. or double-blind:.tw.
62. 60 and 61
