# Supplementary figures and images for: Comparative efficacy and safety of statin and fibrate monotherapy: A systematic review and meta-analysis of head-to-head randomized controlled trials
Source: PLoS One. 2021 Feb 9;16(2):e0246480. doi: 10.1371/journal.pone.0246480 (PMC7872286; doi:10.1371/journal.pone.0246480)

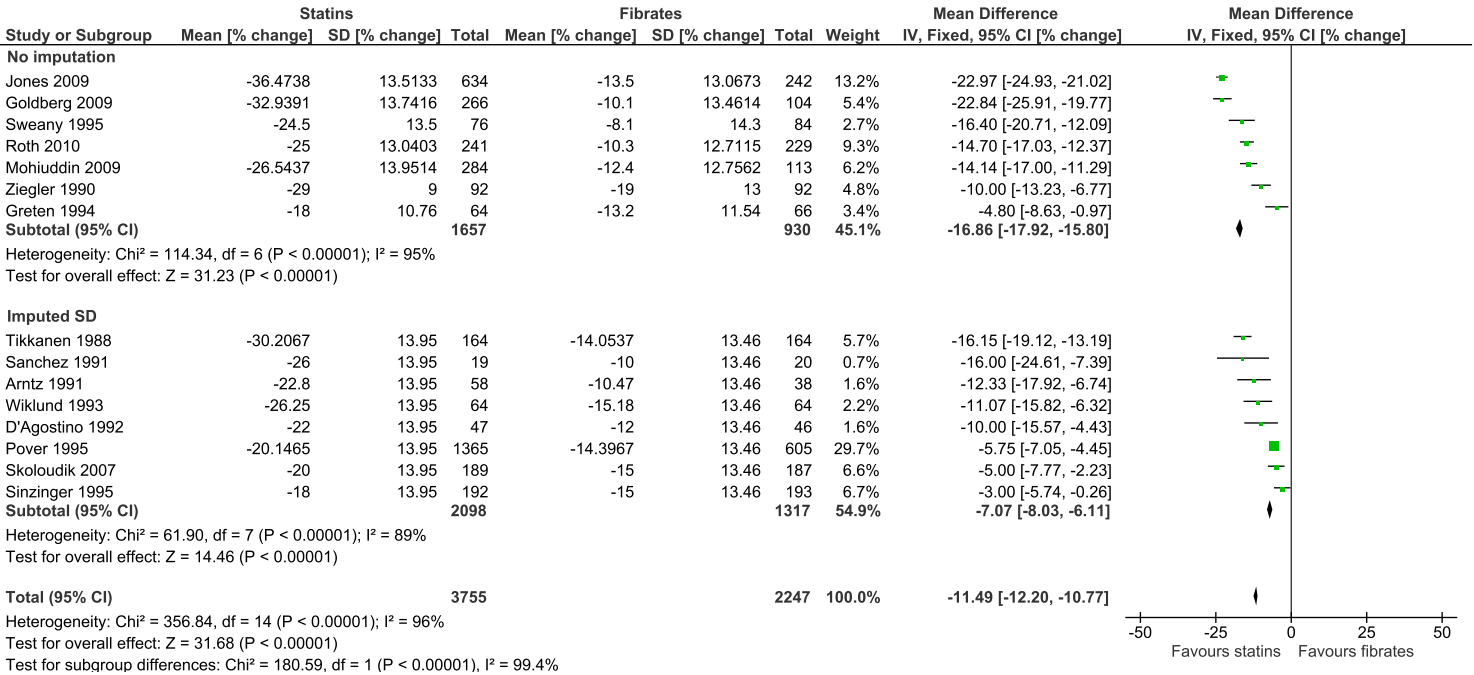

Supplement: S1 Fig — (PDF) [file pone.0246480.s005.pdf]

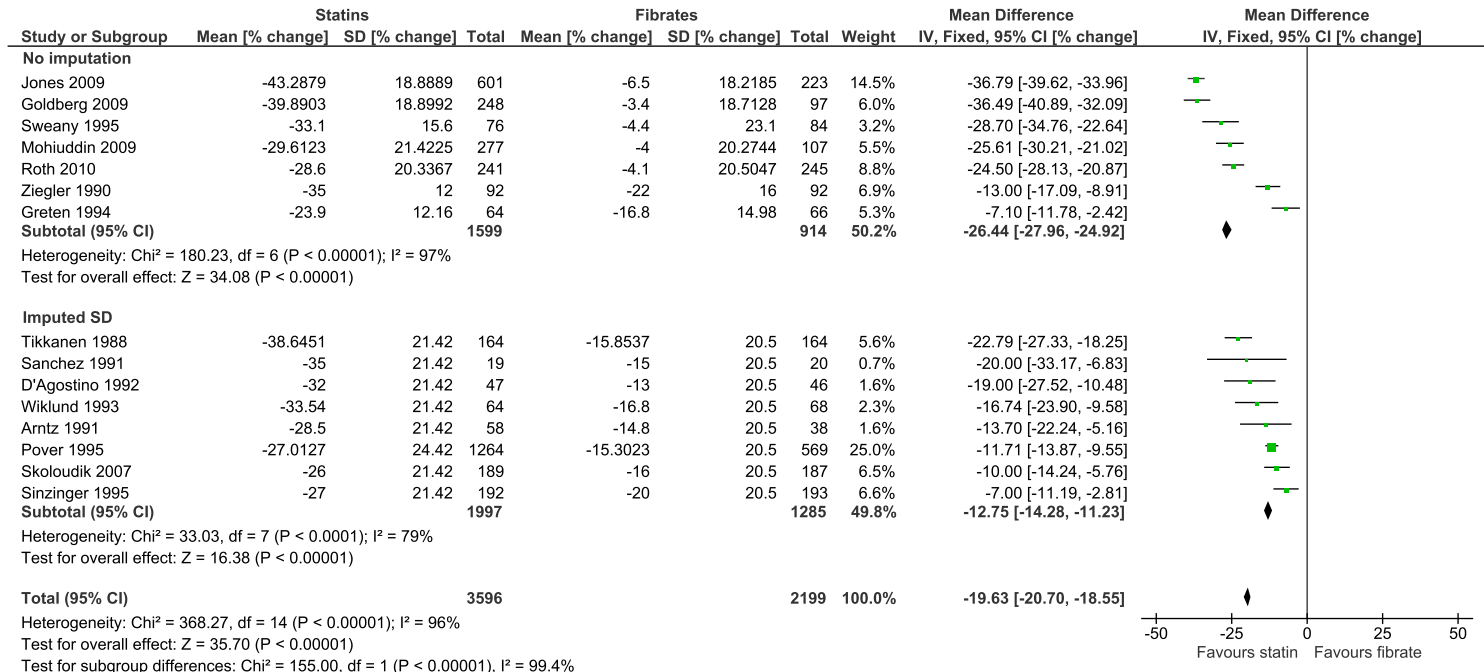

Supplement: S2 Fig — (PDF) [file pone.0246480.s006.pdf]

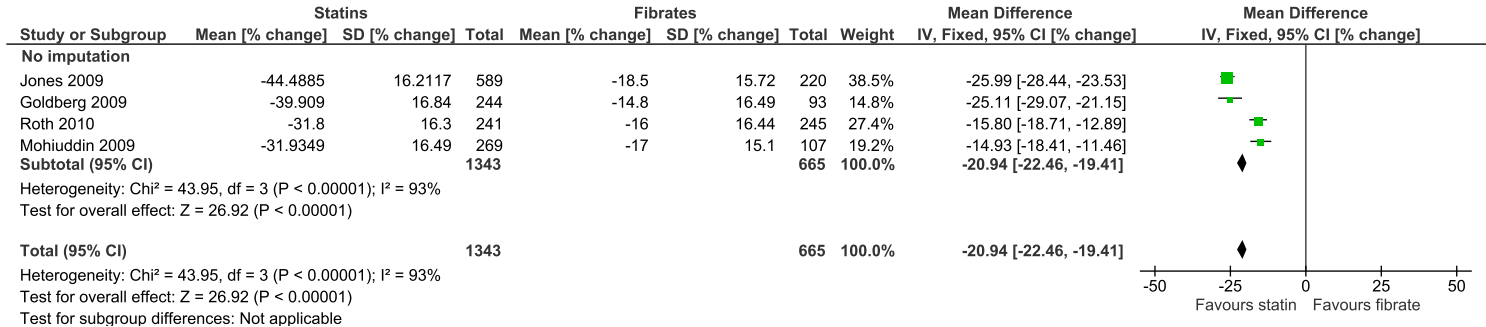

Supplement: S3 Fig — (PDF) [file pone.0246480.s007.pdf]

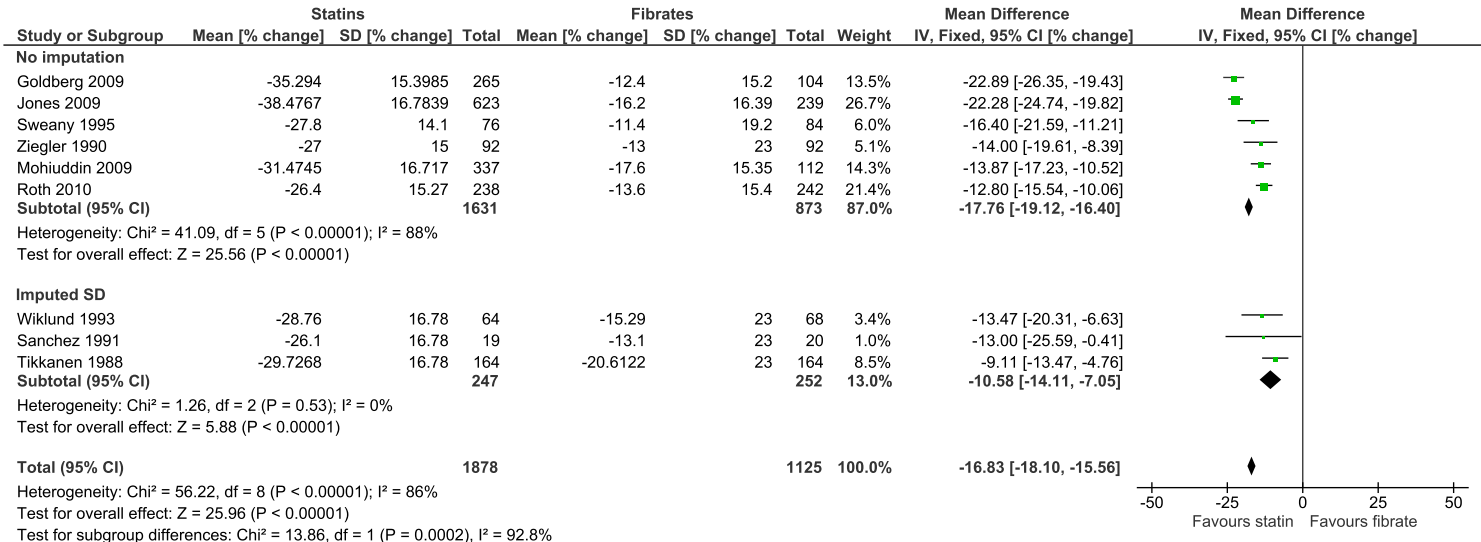

Supplement: S4 Fig — (PDF) [file pone.0246480.s008.pdf]

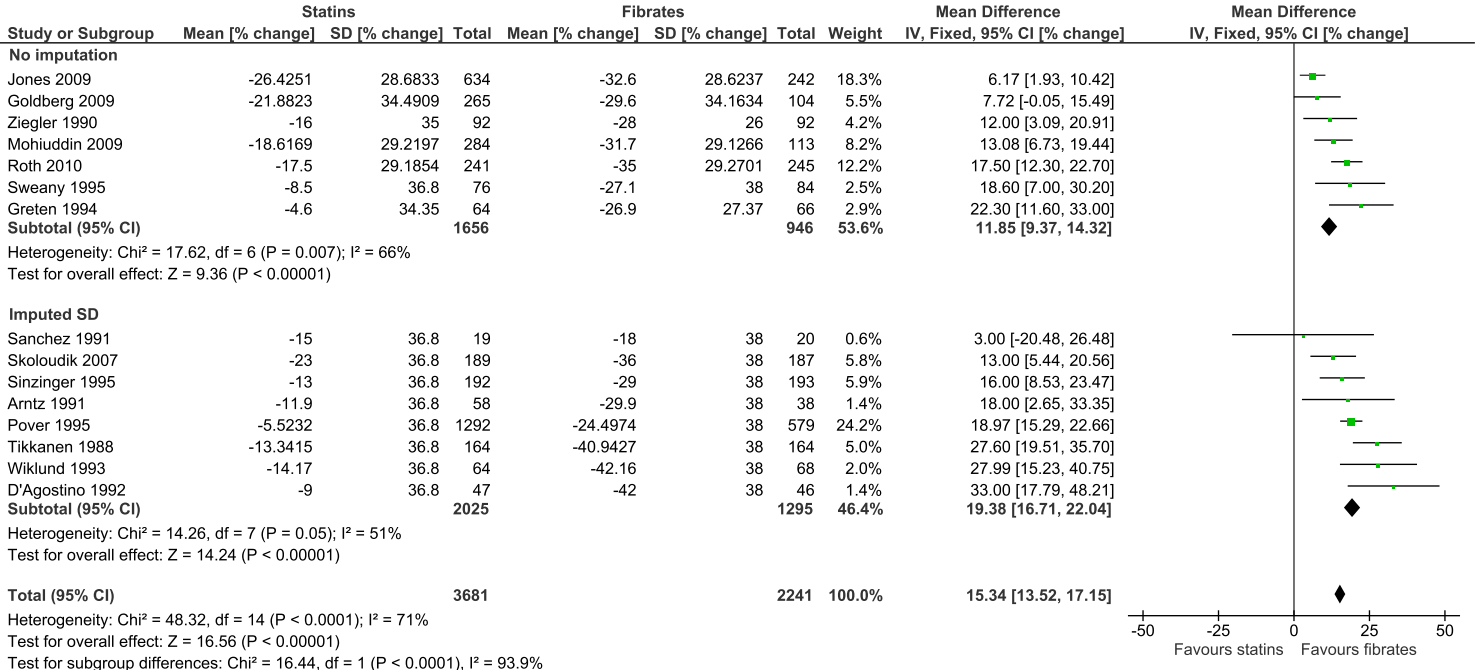

Supplement: S5 Fig — (PDF) [file pone.0246480.s009.pdf]

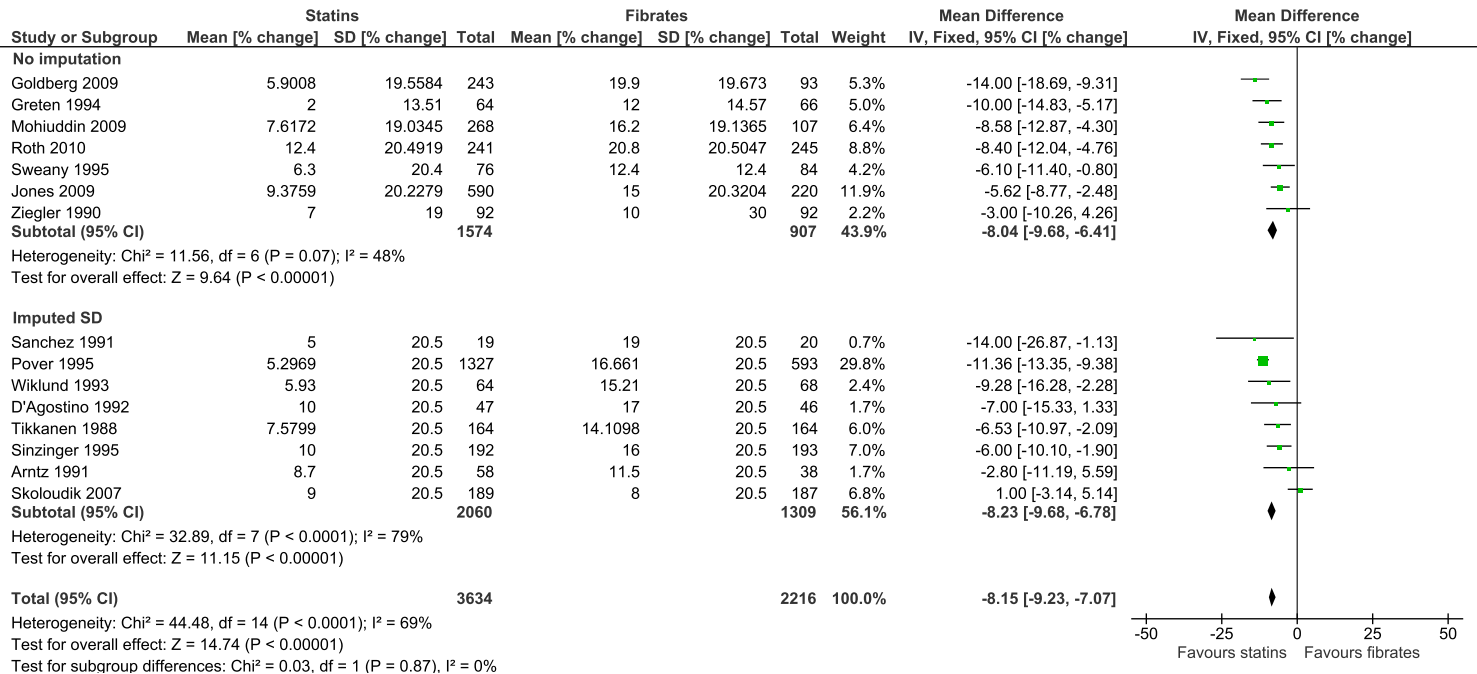

Supplement: S6 Fig — (PDF) [file pone.0246480.s010.pdf]

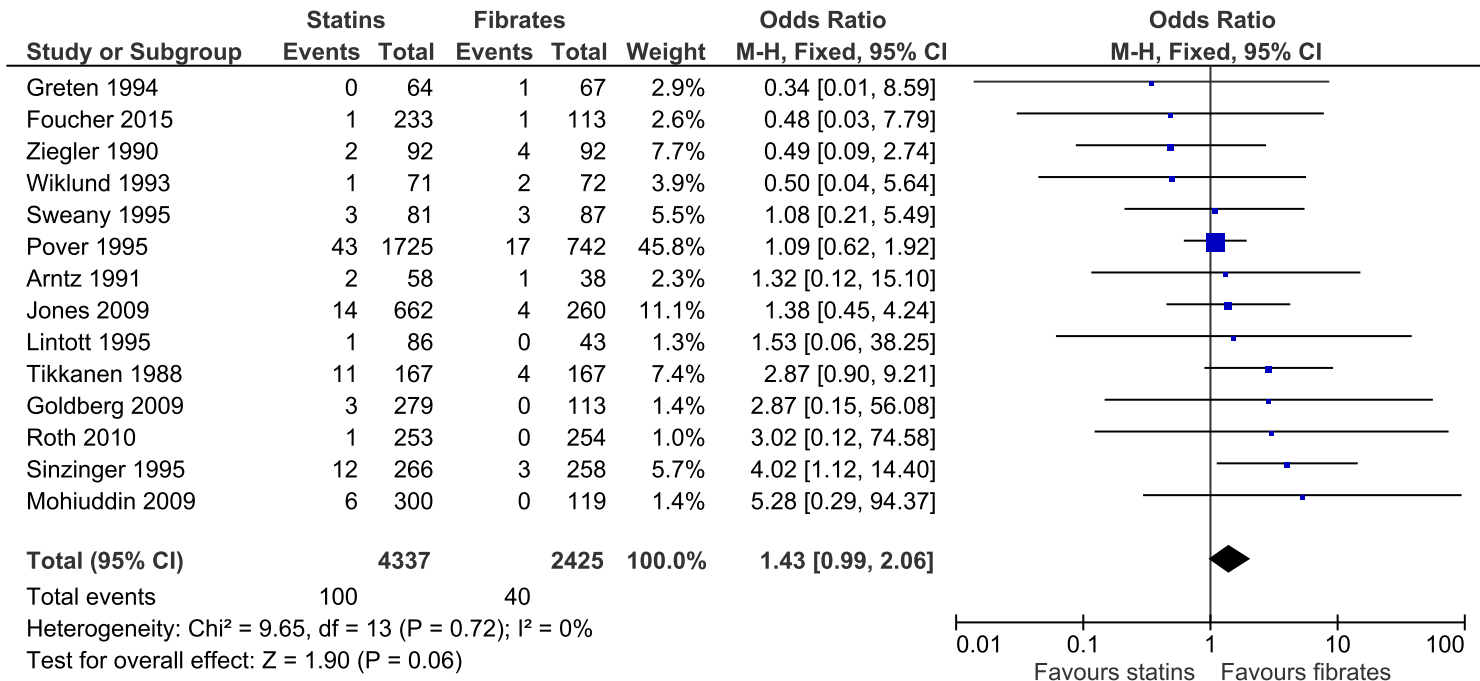

Supplement: S7 Fig — (PDF) [file pone.0246480.s011.pdf]
