# Supplementary material for: Comparative efficacy and safety of statin and fibrate monotherapy: A systematic review and meta-analysis of head-to-head randomized controlled trials
Source: PLoS One. 2021 Feb 9;16(2):e0246480. doi: 10.1371/journal.pone.0246480 (PMC7872286; doi:10.1371/journal.pone.0246480)
Supplement: Attachment — Submitted filename: response_reviewers.docx [file pone.0246480.s012.docx]

**RESPONSE TO EDITOR AND REVIEWER’S COMMENTS**

Thank you for the comments and feedback. They have improved our manuscript. We have addressed each comment and changes in the revised manuscript are indicated by highlights.

**Additional Editor Comments:**

1. **For lines 304-305 remove the negative signs as it could confuse readers.**

Thank you for this suggestion. We have removed the negative signs in lines 304-305 for HDL-C mean differences.

2. **In the Discussion creatinine is misspelled as creatine in at least 2 places.**

Thank you for identifying these spelling errors. We have made the corrections throughout the text.

**Journal Requirements:**

**When submitting your revision, we need you to address these additional requirements.**

**1. Please ensure that your manuscript meets PLOS ONE's style requirements, including those for file naming. The PLOS ONE style templates can be found at**

**https://journals.plos.org/plosone/s/file?id=wjVg/PLOSOne_formatting_sample_main_body.pdf and**

**https://journals.plos.org/plosone/s/file?id=ba62/PLOSOne_formatting_sample_title_authors_affiliations.pdf**

We have reviewed these style requirements and believe our manuscript conforms to all style requirements.

**2. Thank you for stating the following in the Acknowledgments Section of your manuscript:**

**"JEB is supported by the Hong Kong Research Grants Council as a recipient of the Hong Kong PhD Fellowship Scheme."**

**We note that you have provided funding information that is not currently declared in your Funding Statement. However, funding information should not appear in the Acknowledgments section or other areas of your manuscript. We will only publish funding information present in the Funding Statement section of the online submission form.**

**Please remove any funding-related text from the manuscript and let us know how you would like to update your Funding Statement. Currently, your Funding Statement reads as follows:**

**"The author(s) received no specific funding for this work."**

**Please include your amended statements within your cover letter; we will change the online submission form on your behalf.**

We have removed the funding-related text from the Acknowledgments section and have included the revised Funding Statement in the cover letter.

**3. Thank you for stating the following in the Competing Interests section:**

**"I have read the journal's policy and the authors of this manuscript have the following competing interests: EWC has received honorarium from the Hospital Authority, research grants from Research Grants Council (RGC, HKSAR), Research Fund Secretariat of the Food and Health Bureau (HMRF, HKSAR), National Natural Science Fund of China, National Health and Medical research Council NHMRC, Australia), Wellcome Trust, Bayer, Bristol-Myers Squibb, Pfizer, Janssen, Amgen, Takeda, and Narcotics Division of the Security Bureau of HKSAR, outside the submitted work. ICKW has received research grants from Research Grants Council (RGC, Hong Kong), Innovative Medicines Initiative (IMI), Shire, Janssen-Cilag, Eli-Lily, Pfizer, Bayer, Amgen, and grants from European Union FP7 program, outside the submitted work. KYT is an employee of Pfizer Upjohn Hong Kong Limited. The other authors have no competing interests"**

**We note that one or more of the authors are employed by a commercial company: Pfizer Upjohn Hong Kong Limited.**

**3.1. Please provide an amended Funding Statement declaring this commercial affiliation, as well as a statement regarding the Role of Funders in your study. If the funding organization did not play a role in the study design, data collection and analysis, decision to publish, or preparation of the manuscript and only provided financial support in the form of authors' salaries and/or research materials, please review your statements relating to the author contributions, and ensure you have specifically and accurately indicated the role(s) that these authors had in your study. You can update author roles in the Author Contributions section of the online submission form.**

**Please also include the following statement within your amended Funding Statement.**

**“The funder provided support in the form of salaries for authors [insert relevant initials, but did not have any additional role in the study design, data collection and analysis, decision to publish, or preparation of the manuscript. The specific roles of these authors are articulated in the ‘author contributions’ section.”**

**If your commercial affiliation did play a role in your study, please state and explain this role within your updated Funding Statement.**

We have revised the Funding Statement as suggested and included it in our cover letter.

**3.2. Please also provide an updated Competing Interests Statement declaring this commercial affiliation along with any other relevant declarations relating to employment, consultancy, patents, products in development, or marketed products, etc.**

**Within your Competing Interests Statement, please confirm that this commercial affiliation does not alter your adherence to all PLOS ONE policies on sharing data and materials by including the following statement: "This does not alter our adherence to PLOS ONE policies on sharing data and materials.” (as detailed online in our guide for authors http://journals.plos.org/plosone/s/competing-interests) . If this adherence statement is not accurate and there are restrictions on sharing of data and/or materials, please state these. Please note that we cannot proceed with consideration of your article until this information has been declared.**

**Please include both an updated Funding Statement and Competing Interests Statement in your cover letter. We will change the online submission form on your behalf.**

**Please know it is PLOS ONE policy for corresponding authors to declare, on behalf of all authors, all potential competing interests for the purposes of transparency. PLOS defines a competing interest as anything that interferes with, or could reasonably be perceived as interfering with, the full and objective presentation, peer review, editorial decision-making, or publication of research or non-research articles submitted to one of the journals. Competing interests can be financial or non-financial, professional, or personal. Competing interests can arise in relationship to an organization or another person. Please follow this link to our website for more details on competing interests: http://journals.plos.org/plosone/s/competing-interests**

We have included both an updated Funding Statement and Competing Interests Statement in our cover letter that conforms with your recommendations.

**Reviewers' comments:**

**Reviewer's Responses to Questions**

**Comments to the Author**

**1. Is the manuscript technically sound, and do the data support the conclusions?**

**The manuscript must describe a technically sound piece of scientific research with data that supports the conclusions. Experiments must have been conducted rigorously, with appropriate controls, replication, and sample sizes. The conclusions must be drawn appropriately based on the data presented.**

**Reviewer #1: Partly**

**Reviewer #2: Partly**

**2. Has the statistical analysis been performed appropriately and rigorously?**

**Reviewer #1: Yes**

**Reviewer #2: Yes**

**3. Have the authors made all data underlying the findings in their manuscript fully available?**

**The PLOS Data policy requires authors to make all data underlying the findings described in their manuscript fully available without restriction, with rare exception (please refer to the Data Availability Statement in the manuscript PDF file). The data should be provided as part of the manuscript or its supporting information, or deposited to a public repository. For example, in addition to summary statistics, the data points behind means, medians and variance measures should be available. If there are restrictions on publicly sharing data—e.g. participant privacy or use of data from a third party—those must be specified.**

**Reviewer #1: Yes**

**Reviewer #2: Yes**

**4. Is the manuscript presented in an intelligible fashion and written in standard English?**

**PLOS ONE does not copyedit accepted manuscripts, so the language in submitted articles must be clear, correct, and unambiguous. Any typographical or grammatical errors should be corrected at revision, so please note any specific errors here.**

**Reviewer #1: Yes**

**Reviewer #2: Yes**

**5. Review Comments to the Author**

**Please use the space provided to explain your answers to the questions above. You may also include additional comments for the author, including concerns about dual publication, research ethics, or publication ethics. (Please upload your review as an attachment if it exceeds 20,000 characters)**

**Reviewer #1:**

**1. Is the manuscript technically sound, and do the data support the conclusions? Statins versus fibrates OR of 0.17 should report the results as a decrease not an elevation of serum creatinine due to statins**

Thank you for reviewing our manuscript and for this comment. The outcome of interest is elevation in serum creatinine and we apologize for the unclear reporting in lines 338-340 where we wrote “Statins greatly reduced the risk of elevations in serum creatinine (OR 0.17, 95% CI 0.08–0.36, I^2^=0%; six studies, n=2553; high certainty; Fig 13).” We have revised this sentence to “Statins greatly reduced the risk of elevated serum creatinine…” which should improve clarity of reporting this outcome (line 340).

**Reviewer #2:**

**Statins are first-line agents for hypercholesterolemia. Current guidelines do not recommend the use of fibrates as alternative to statins in the absence of hypertriglyceridemia. However, fibrates have the labeled indication of hypercholesterolemia or mixed dyslipidemia when statins are contraindicated or not tolerated. Therefore, the relative efficacy of fibrates versus statins in clinically relevant outcomes is an interesting question.**

**The review uses the Cochrane method. After looking for randomized controlled trials that directly compare statins with fibrates, authors find very little evidence about their relative efficacy. Evidence for safety is more robust, favoring statins in most outcomes.**

**Methods are sound but the reporting has to be improved. Some statements do not correspond with the findings. Definition of secondary does not correspond with clinical practice. There are some inconsistence in the assessment of the quality of evidence. Some data need revision. Authors should rewrite some sentences to avoid confusion. Discussion has to be improved. There are some mistakes.**

**The article needs several changes prior to being published.**

Thank you for your detailed review and comments. They have enhanced our manuscript.

**Major issues**

**Statements that are not based in findings**

1. **Line 45 “…but might be associated with an increased risk of myalgia (OR 1.32, 95% CI 0.95–1.83…” since there is no statistically significant difference in the risk of myalgia, the sentence has to be rewritten to say that there was no clear evidence to support a difference in the risk of myalgia.**

We have revised the Abstract to better align with the interpretation of the statistical estimates as described in our text. We have revised the Abstract results as suggested by the reviewer: “We did not find any eligible evidence of a difference for statins versus fibrates for cardiovascular mortality (OR 2.35, 95% CI 0.94–5.86, I^2^=0%; ten studies, n=2657; low certainty), major cardiovascular events (OR 1.15, 95% CI 0.80–1.65, I^2^=13%; 19 studies, n=7619; low certainty), and myalgia (OR 1.32, 95% CI 0.95–1.83, I^2^=0%; ten studies, n=6090; low certainty).”

2. **Line 330. “the relevance of elevations in CK, which tended to favour fibrate monotherapy (OR 1.43, 95% CI 0.99–2.06,” is not an evidence-based statement; the confidence interval includes no difference.**

We agree with the reviewer and have revised this sentence to “There was no clear evidence of a difference for myalgia (OR 1.32, 95% CI 0.95–1.83, I^2^=0%; ten studies, n=6090; low certainty; Fig 11) or for elevations in CK (OR 1.43, 95% CI 0.99–2.06, I^2^=0%; 14 studies, n=6762; S7 Fig).”

3. **Line 39. “We did not find any eligible evidence of a difference” As some evidence has been selected, it would be more appropriate to say… not find evidence of a difference…**

Thank you for this comment. We have found it challenging to accurately report the magnitude of difference of the evidence between the two treatments that is most appropriate. Indeed, earlier versions of our manuscript used similar wording as suggested by the reviewer (“claims of no difference”). However, we have now deliberately avoided using “statistically significant” or any claims of “no difference” or “no effect” and have attempted to adopt the suggested wording of Marston and colleagues: “we did not find any eligible evidence of a difference”.[1]

4. **Lines 66-68. This is a more accurate statement "To date, systematic reviews of fibrates have made indirect comparisons, usually contrasting the intervention of interest with placebo or usual care and have excluded head-to head comparisons [5-10] "**

We have revised these lines as suggested by the reviewer.

**Definition that does not correspond with clinical practice**

5. **Line 173. “studies with more than 10% of participants with baseline cardiovascular disease were defined as secondary prevention”. This definition is misleading. A study with only 18% of patients with baseline cardiovascular disease are not equivalent to a secondary prevention population in which all patients have cardiovascular disease. The authors should change this designation.**

We selected the 10% threshold since it matches the definition for primary prevention studies previously selected in the Cochrane review of fibrates for primary prevention that we cited.[2] We have indicated in the methods that this cut-point is arbitrary, and are aware that it does not match the definition used in clinical practice. Typically, patients without a prior history of atherosclerotic cardiovascular disease are considered primary prevention patients.

**Inconsistence in the assessment of the quality of evidence**

6. **The certainty of evidence for cardiovascular mortality is downgraded for allocation concealment, incomplete outcome data, blinding of outcome assessment, and selective reporting in several studies. It is not clear why these problems do not affect others outcomes. Moreover, the study Pover 1995, with high risk of bias on these items, is excluded from the analysis of cardiovascular mortality and included in those of all-cause mortality and major cardiovascular events.**
   We have discussed our judgement for the outcomes and have decided to downgrade the certainty of evidence for all-cause mortality, serious adverse effects, and myalgia, for risk of serious bias, as suggested by the reviewer. Judgements of the certainty of evidence for these outcomes have been revised throughout the text and supplementary material. The difficulty with Pover 1995 is that the authors only report the number of patients who died in each group, and that 80% of all deaths were from myocardial infarction (included in our outcome definition of cardiovascular mortality). However, Pover 1995 did not report the number of patients in each treatment group that died from myocardial infarction. Therefore, this study was excluded from the cardiovascular mortality analysis.
7. **Line 238. A reason for high risk of bias is blinding of outcome assessment for subjective outcomes. Myalgia is the more subjective outcome. In spite of most of the events coming from studies with high risk of bias, evidence was rated as moderate. Downgrading of evidence must be consistent.**

As described in point #6, we agree with the reviewer and have attempted to adopt a more consistent approach to assessing the certainty of the evidence. Myalgia has been downgraded to low certainty of evidence.

**Confusing sentences**

8. **Line 44. “.. and elevations in serum creatinine (OR 0.17…” is confusing. It should be with reduced risk of elevation in serum creatinine.**

Thank you for identifying this problematic sentence and for the suggested wording. We have revised the Abstract so that it now reads “…reduced the risk of elevations in serum creatinine”.

9. **Line 47. As there is a dichotomous outcome it would be better to say: and might be associated with an increased risk of elevation in alanine aminotransferase**

We have revised the sentence similar to what was suggested (line 47): “…and might increase the risk of elevations in alanine aminotransferase.”

10. **“Primary prevention studies were arbitrarily defined as those which enrolled participants with a baseline history of cardiovascular disease of 10% or less,” this phrase needs rewording**
    Please see our response to point #5. We think that the sentence is clear, but are open to suggestions on how to revise it to enhance clarity for the reader. We defined a study to be in the primary prevention subgroup if the proportion of all the patients in the study with a history of cardiovascular disease was ≤ 10%. As summarized in Table 1, some of the studies do not report a history of cardiovascular disease for the trial participants.
11. **Please see Line 278. “Pooling eligible studies suggests statins and fibrates for major cardiovascular events” does not make sense. I think you mean ....are not different in their effect on major cardiovascular events"**
    We apologize for this error. This sentence has been corrected and now reads “In the eligible studies, no evidence of a difference for statins and fibrates was observed for major cardiovascular events.”
12. **Line 188. “To assess the validity of imputing standard deviations for the continuous outcomes, we separated studies requiring imputation of standard deviations into a subgroup.” The validity of imputing standard deviations is assumed. It is not clear how they can be assessed in this way. Presenting as subgroups can be a way to inform the reader in what studies the imputation was done.**
    Thank you for this important comment. We agree with the reviewer and have revised the sentence (line 188) as follows: “To show the effect of imputing standard deviations for the continuous outcomes, we separated studies requiring imputation of standard deviations into a subgroup in the forest plots.”
13. **In the forest plots, it is not clear why SD are different in studies to which SD have been imputed.**
    We suspect this may have been caused by slight rounding differences or data entry errors in the RevMan software. We have now ensured that the imputed SDs are all equal for each continuous outcome. The interpretation of the estimates is unchanged but there are some slight numerical changes. The relevant supplementary figures have been revised (S1, S2, S4, S5, S6).

**Data that need review**

14. **Line 62. The source of unpublished data should be reported.**

We have revised this line and now cite our unpublished manuscript that reports global consumption trends of lipid-modifying drugs, including statins and fibrates. This paper uses data from IQVIA MIDAS and provides the most recent and comprehensive information on global consumption of lipid-modifying drugs. Therefore, the reference to unpublished data from Hong Kong has been deleted.

15. **Line 185. Ref 22 does not fit with the statement.**
    We have corrected this error and cited the intended paper.
16. **Line 187. I cannot find information about the Peto method in GRADEpro [23].**
    We apologize for the incorrect citation. We have corrected this error and cited the intended paper which discusses the appropriateness of the Peto method.

**Table 1.**

17. **Sinzinger 1995: It is not clear how the 18% of participants with CAD disease has been calculated. The article only provided data of per-protocol population. It seems to be 109 patients with CAD disease (myocardial infarction, angina or coronary bypass/PTCA)**
    Thank you for the question. Table 1 of Sinzinger 1995 separately reported a previous history of MI (bezafibrabe: 15 patients; lovastatin: 15 patients; total: 30), angina (b: 36; l: 34; total: 70), and coronary bypass/PTCA (b: 5; l: 4; total: 9). Because a single patient could have both a history of MI and angina, for example, we decided to use the highest reported percentage for angina for the baseline history of cardiovascular disease. This number was calculated as 70/385 (192+193 patients in each treatment arm) = 0.18. To be clearer to the reader we have changed the cell entry for this study from “Secondary

(CAD: 18%)” to “Secondary (Angina: 18%)”.

18. **Goldberg 2009, Jones 2009, Mohiuddin 2009, Roth 2010: Treatment period is 12 weeks**
    Thank you for raising this point. Indeed, these studies had a treatment period of 12 weeks. However, they also had a 30-day safety follow-up period after the end of treatment. For example, the study duration from Goldberg 2009 is reported as follows “The study duration was approximately 22 weeks, with a 6-week diet run-in period (during which patients underwent wash out of any previous lipid-lowering medications), a 12-week treatment period, and a 30-day safety follow-up period.” Since we also included safety outcomes in our review, we report the total duration of follow-up for each of these studies, which was the treatment period plus the safety period (about 16 weeks).
19. **Fig 1. Flowchart: there is a mistake. The branch “5 records identified through other sources” has to be higher than “360 full-text..”. It would be noted 19 studies (coming from 24 articles); otherwise, numbers are inconsistent.**
    We agree that the final numbers do not precisely sum in Fig 1. However, Fig 1. is an accurate description of our study process. The reason for the discrepancy is because the boxes change from reporting the number of articles to the number of studies. As reported in the results line 211 “Nineteen studies (reported in 24 articles) met the inclusion criteria”.
20. **Line 272. Results of all-cause mortality in the text and in Fig 3 are different.**
    Thank you for pointing out this error. We have checked our analysis and have revised the text with the correct results from Fig 3 (line 274).
21. **Line 299. Results of LDL-C in the text and in S2Fig are different.**

Thank you for pointing out this error. We have checked our analysis and figure. We apologize for mistakenly exporting the incorrect figure of our sensitivity analysis (random effects model). The figure has been corrected to the fixed effects model and now matches the results in the text.

22. **In Sano 2010, the number of major cardiovascular events is 12 in the statin group.**
    Thank you for pointing out this error, we have changed this to 12 events for the statin group. Updated estimates and forest plots and estimates for this outcome are included in the Abstract (line 43), the Results (line 281), Figure 4, and the Supporting Information (Figure 12).

**Issues with Discussion**

23. **Line 421: “Our included studies identified a greater number of total myalgia events than other comprehensive fibrate reviews.[7, 10] This may be because ascertainment of muscle related adverse effects may have been more deliberate in our included studies as these are known adverse effects of statins and fibrates, and adverse effects may have been assessed more systematically than in larger placebo controlled studies.” This is very speculative. Others reviews have a different scope and include different studies with placebo groups.**

We agree with the reviewer and have revised lines 425-432 by removing this speculative comment. The revised section now states: “Our included studies identified a greater number of total myalgia events than other comprehensive fibrate reviews.[5, 8] Despite including a larger number of myalgia events in this study, our estimates for myalgia remained uncertain, with the bounds of the confidence interval including both a potential reduction and an increase in risk. Determining the true incidence and risk factors for statin associated muscle-related adverse effects remains an active area of research.”

24. **The PROMINENT study do not compare directly statins with fibrates, so it is not relevant for the discussion.**

The reviewer is correct and we have removed this point from the discussion. Please see lines 450-453 for the revised paragraph discussing the ongoing PIONEER study.

25. **Line 404. “The short duration of study follow-up, and perhaps a similar anticipated reduction in cardiovascular events, explain why we could not detect a difference in clinical efficacy outcomes”. Author should clarify this statement. Does it mean that the results are consistent with those of the previous indirect comparisons?**
    We have clarified and expanded this statement. The revised sentence is in the Discussion lines 406-413 and reads as follows: “The lack of evidence of a difference between statins and fibrates in this study is consistent with the similar relative benefit observed in indirect comparisons. However, it is more likely a result of the short duration of follow-up and small number of outcome events, since the time to benefit in large-scale trials of lipid-lowering medications is typically greater than one year.[47] Therefore, detecting a difference in efficacy between active treatments, if one exists, would require a much larger sample size and a longer duration of follow-up than is available from the eligible trials.”

26. **In order to put the review in the context of current clinical practice, it would be helpful to note that statins and doses used in the included trials are not of high intensity treatment currently recommended for secondary prevention.**
    Thank you for the suggestion. We have added this point to the Discussion (line 441): “Lastly, in the context of secondary prevention patients, the choice of statin drug and statin dose intensity in many of the eligible trials does not align with current guideline recommendations for treatment with high-intensity statins.”

**Minor issues**

27. **The authors should remark that the technical name of statins is HMG CoA reductase inhibitors.**

Thank you for the suggestion, but we prefer to use the term statins throughout the text for consistency. However, we have added HMG CoA reductase inhibitors as a keyword to facilitate indexing under the technical name of statins.

28. **Line 179. Finding only one study that met the definition of diabetes is a result.**
    We agree, and have moved this sentence to the Results. The revised sentence (line 224) now reads “Only one study met our pre-specified definition of enrolling participants with diabetes mellitus, thus we did not conduct this subgroup analysis.”
29. **Line 58-60: I would like the authors clarify the period in which the use of fenofibrate has been evaluated. References 1-4 provide quite old data. Is there current information?**
    The reviewer is correct, and we have removed these older references. We have recently completed a study using global pharmaceutical sales data, that to our knowledge, has the most current information on global use of lipid drugs, including fibrates. We have cited the preprint of this manuscript in the Introduction section (lines 61-63) and specify the dates for the included data, 2008-2018.
30. **Line 72. “In addition, we examined tolerability and safety outcomes for the included studies”. Tolerability and safety outcomes have to be analysed according to the methodology. Re-write the sentence. The assessment of safety is critical in a systematic review about medicines. The author should re-write the sentence.**

We agree with the reviewer that an assessment of safety is critical for systematic reviews of medicines. Our primary objective was to assess efficacy, while our secondary objective was to assess the safety and tolerability in the eligible studies. We have attempted to convey this to the reader in the revised line 73, which now reads “Our secondary objective was to assess the comparative tolerability and safety of statins and fibrates in the eligible head-to-head studies.”

**References**

1.	Marson Smith PR, Ware L, Adams C, Chalmers I. Claims of 'no difference' or 'no effect' in Cochrane and other systematic reviews. BMJ Evidence-Based Medicine. 2020:bmjebm-2019. Epub 2020/01/09. doi: 10.1136/bmjebm-2019-111257. PubMed PMID: 31911421.

2.	Jakob T, Nordmann AJ, Schandelmaier S, Ferreira-González I, Briel M. Fibrates for primary prevention of cardiovascular disease events. Cochrane Database Syst Rev. 2016;(11). doi: 10.1002/14651858.CD009753.pub2. PubMed PMID: CD009753.
